# Exploration of novel hydroxamic acid-based candidates integrating chalcone scaffold cap as multitarget HDAC inhibitors: design, anti-prostatic cancer assessment, *in silico* docking and molecular dynamic simulation

**DOI:** 10.1080/14756366.2026.2711875

**Published:** 2026-08-27

**Authors:** Aya I. Abd El-hameed, Ibrahim M. Salem, Rasha M. Allam, Mariem M. Arafat, Hossameldin A. Aziz, Zuhier A. Awan, Tarek S. Ibrahim, Omar Alshazly, Eman A. M. Beshr, Stefan Bräse, Mamdouh F. A. Mohamed

**Affiliations:** a Depatment of Pharmaceutical Chemistry, Faculty of Pharmacy, Sohag University, Sohag, Egypt; b Department of Pharmaceutical Chemistry, Faculty of Pharmacy, Sphinx University, New Assuit City, Assiut, Egypt; c Pharmacology Department, Medical and Clinical Research Institute, National Research Centre, Dokki, Cairo, Egypt; d Cell Culture Unit, Life Science Division, Nawah-Scientific Inc, Cairo, Egypt; e Department of Pharmaceutical Chemistry, Faculty of Pharmacy, New Valley University, New Valley, Egypt; f Department of Pharmaceutical Chemistry, Faculty of Pharmacy, New Valley National University, New Valley, Egypt; g Department of Clinical Biochemistry, Faculty of Medicine, King Abdulaziz University, Jeddah, Saudi Arabia; h Department of Organic Chemistry, Faculty of Pharmacy, King Abdulaziz University, Jeddah, Saudi Arabia; i Department of Medicinal Chemistry, Faculty of Pharmacy, Minia University, Minia, Egypt; j Medicinal Chemistry Department, Faculty of Pharmacy, Minia National University, New Minia, Egypt; k Institute for Biological and Chemical System, Karlsruhe Institute of Technology, Karlsruhe, Germany

**Keywords:** Chalcone, HDAC inhibitors, targeted anticancer, prostate cancer, osteosarcoma

## Abstract

Two novel series of hydroxamic acid hybrids were designed and synthesised as anticancer agents *via* histone deacetylase isozymes (HDACs) inhibitors. Antiproliferative effects of novel compounds were evaluated against DU-145 and PC-3 (prostate cancer) and MG-63 (osteosarcoma) cell lines. Compound **5d** emerged as the most potent against DU-145 and PC-3 (prostate cancer) with IC_50_ values of 0.93 and 1.05 µM, exhibiting inhibiting HDAC 2, 4, 6, and 8 with IC_50_ values of 0.86, 0.17, 1.17, and 0.15 µM respectively. As a result, it was selected for mechanistic investigations. Apoptotic cell death modality was predominantly detected in the three cell lines, accompanied by cell cycle arrest in the S phase in prostate cancer cell lines, whereas it caused cell cycle arrest in MG-63 osteosarcoma cells in the G0/G1 phase. Docking studies confirmed its high affinity for HDACs, and an atomistic standard 100 ns dynamic simulation supported the stability of these interactions. In conclusion, all these findings suggested the potential use of this compound for treating prostate cancer with bone metastasis.

## Graphical abstract

**Figure:**
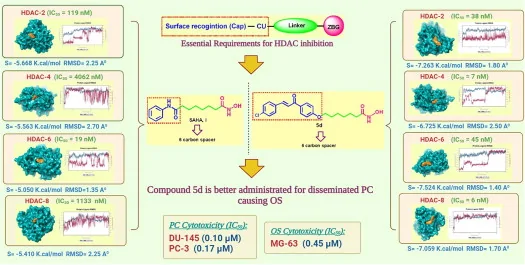

## Introduction

Prostate cancer (PC) ranks as the second most frequently diagnosed malignancy and the third leading cause of cancer-related mortality among men all over the world. PC represents a biologically heterogeneous disease, with an aetiology influenced by a multifaceted array of factors, including lifestyle behaviours, inherited genetic variations, and epigenetic modifications^1^. PC cases exhibit an age-dependent increase in both incidence and mortality, with a mean age onset of around 66 years. However, the epidemiological data highlight a significant racial disparity; for example, African American men display markedly elevated PC incidence rates of 158.3 cases per 100,000 which is nearly double the mortality rate compared to White men. These disparities may reflect underlying differences in molecular pathogenesis, including variations in genomic, transcriptomic, and epigenetic profiles^2^. Despite the success of treatment for localised and recurrent PC cases, most patients develop castration resistant prostate cancer (CRPC), an advanced form of prostate cancer with aggressive characteristics and high proliferation and metastasis rates, resulting in a poor prognosis, no curative therapeutic options, and overall reduced survival rates^3^. Besides, regarding the seed and soil theory, where neoplasm metastasis is an organ-specific phenomenon influenced by the interactions linking disseminating tumour cells as seeds and the host organs as soil^4^, more than 90% of patients diagnosed with advanced PC show evidence of bone metastasis, especially causing remarkable osteosarcoma^5–8^. Osteosarcoma (OS) is the most frequent type of bone cancer, accounting for 20–40% of all bone malignancy cases^9^. Furthermore, people with a localised OS had an 80% five-year overall survival rate, whereas patients with metastatic bone cancer, on the other hand, have an even poorer survival rate^10^.

The most extensively used *in vitro* cell lines for prostate cancer research are the Du-145 and PC-3, derived from metastatic deposits representing CRPC, which justify the reported prevalence of the illness in humans^11^. Therefore, they are valuable models for studying advanced PC cases^12^. While the PC3 cell line has the characteristics of late-stage aggressive prostate cancer, the DU145 cell line outperforms it in predicting clinical effects in human subjects^13^.

In parallel, extensive research efforts have been directed towards the development of molecularly targeted agents, with the primary objective of achieving superior anticancer efficacy while minimising associated adverse effects^14^. The emergence of many medications that specifically target enzymes involved in the epigenetic regulation of gene expression positions epigenetic targets as a promising and valuable strategy for both chemotherapy and chemoprevention of cancer^15^^,^^16^.

Histone deacetylase enzymes (HDACs) accomplish an important role in cancer propagation through an elimination of the ε-*N*-acetyl groups from the lysine residues on both histone and non-histone proteins, thereby modulating chromatin dynamics and transcriptional regulation^17^. The deacetylation of the lysine residues on histone proteins results in transcriptional repression by promoting a more compact chromatin structure, which limits the accessibility of transcription factors to DNA. In this condensed, transcriptionally inactive chromatin state, genes involved in cell cycle arrest and apoptosis such as p21^WAF1/CIP1^, Gadd45, FAS, and caspase-3 are stopped, contributing to the survival and progression of tumour cells^18^. Thus, the inhibition of HDACs leads to histone hyperacetylation, resulting in a more open chromatin structure that facilitates the induction of differentiation, cell cycle arrest, and apoptosis in cancer cells. An aberrant activity and overexpression of HDACs have been confirmed in a variety of human cancer types, such as ovarian, colon, breast, and prostatic cancers, along with lymphoma and osteosarcoma^19^^,^^20^. Consequently, HDAC inhibitors constitute a potential category of targeted anticancer medicines, demonstrating considerable efficacy against a wide range of neoplasms, at levels that are well tolerated by patients with cancer^21^^,^^22^.

**Figure 1.**
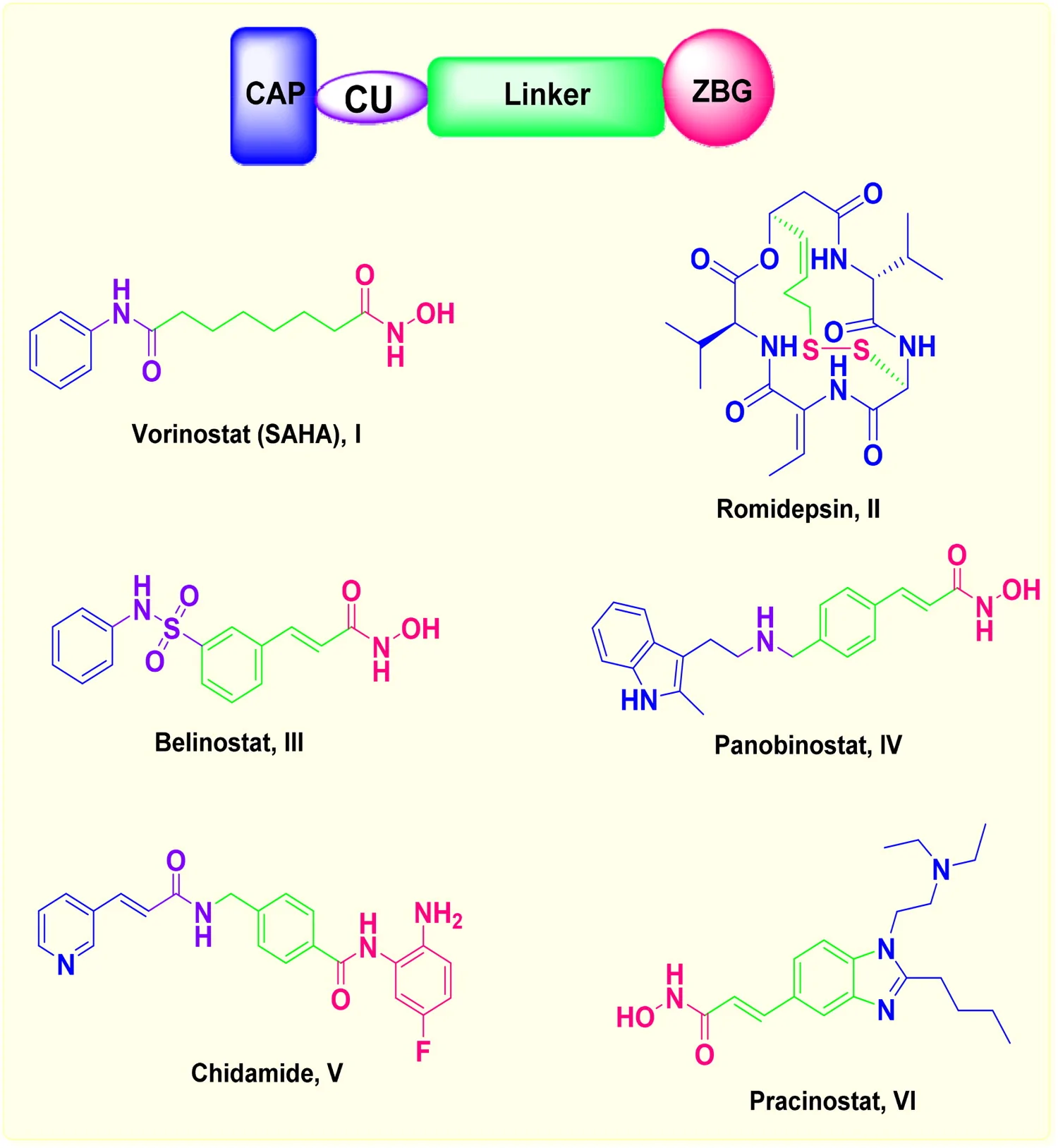
Pharmacophoric features and structures of the approved HDAC inhibitors.

To date, six HDAC inhibitors have been approved for the treatment of cutaneous T-cell lymphoma, involving Vorinostat (SAHA) **I**, Romidepsin **II**, Belinostat **III**, Panobinostat **IV**, Chidamide **V,** and Pracinostat **VI** (Figure 1). The common structural features of all HDAC is are; i) zinc-binding group (ZBG) able to coordinate the Zn^2+^ cofactor at the bottom of the enzyme cavity (mainly acid hydroxamate); ii) a cap group (CAP) allowing to interact with the rim of the catalytic tunnel of the enzyme; iii) a polar connection unit; iv) hydrophobic linker or spacer to enable the inhibitor to lie into the catalytic tunnel of the enzyme^23^^,^^24^.

**Figure 2.**
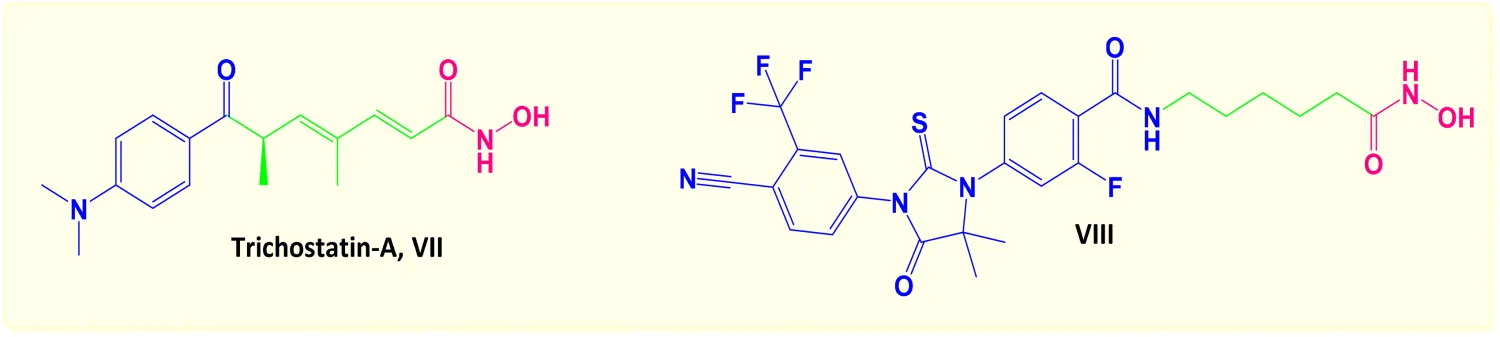
Representative examples of reported hydroxamic acid-based HDAC inhibitors used in the management of resistant prostate cancer.

Research on the therapeutic potential of HDAC inhibitors in prostate cancer is limited. The inhibition of isoenzymes HDAC2, HDAC4, HDAC6, and HDAC8 demonstrated a significant impact, particularly in treatment-resistant prostate cancer, where these isoenzymes were overexpressed^25–29^. The hydroxamate-derived compound SAHA (**I**) exhibited growth inhibitory activity in resistant prostate cancer cell lines at micromolar concentrations (2.5–7.5 µM)^30^^,^^31^. Similarly, trichostatin A (VII), a compound derived from hydroxamate, impeded cellular invasion and migration by downregulating the expression of the Slug gene *via* HDAC-2 inhibition^32^. The thiohydantoin-hydroxamate hybrid (**VIII**), connected by a hydrocarbon spacer, demonstrated dual inhibition of HDAC-6 (IC_50_ = 0.0356 µM) and antagonism of the androgen receptor, successfully addressing resistant prostate cancer^33^ (Figure 2).

**Figure 3.**
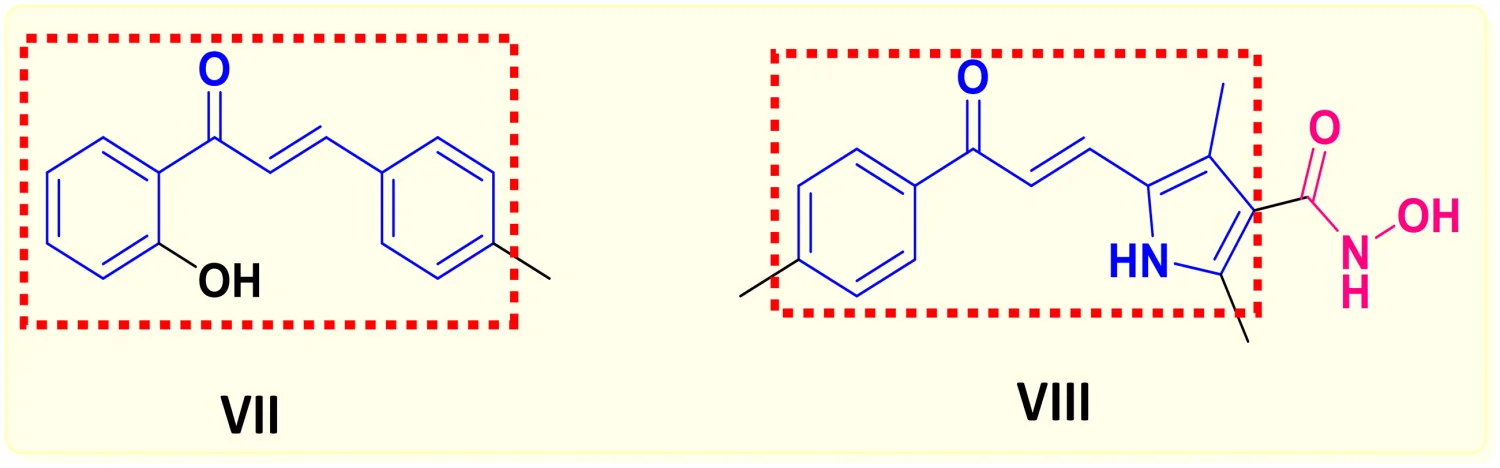
Chemical structure of basic chalcone **VII**, and the chalcone bearing hydroxamic acid **VIII**, as HDAC inhibitors.

Moreover, chalcones (1,3-diaryl-2-propen-1-ones) constitute another important class of natural products belonging to the flavonoid family, which displayed interesting diverse biological investigations^25–30^. Interestingly, substituted chalcones were tested and screened for their potential cytotoxic activity against various cancer types^31–34^. Providentially, the basic chalcone skeleton was proven for its HDAC inhibitory potential, such as the 2-hydroxy chalcone compound **VII** that revealed substantial cytotoxicity against different colon and renal cell lines with HDAC inhibition (IC_50_ = 105.05 µM)^35^. In addition, the conjugation of the chalcone structure as a cap with the hydroxamic acid moiety as ZBG in compound **VIII** caused decent elevation of HDAC1 inhibition potential (IC_50_ = 0.14 µM) comparable to SAHA (IC_50_ = 0.38 μM)^36^, which discloses the pharmacophoric significance of the chalcone scaffold regarding HDAC inhibition potential **(**Figure 3**)**.

**Figure 4.**
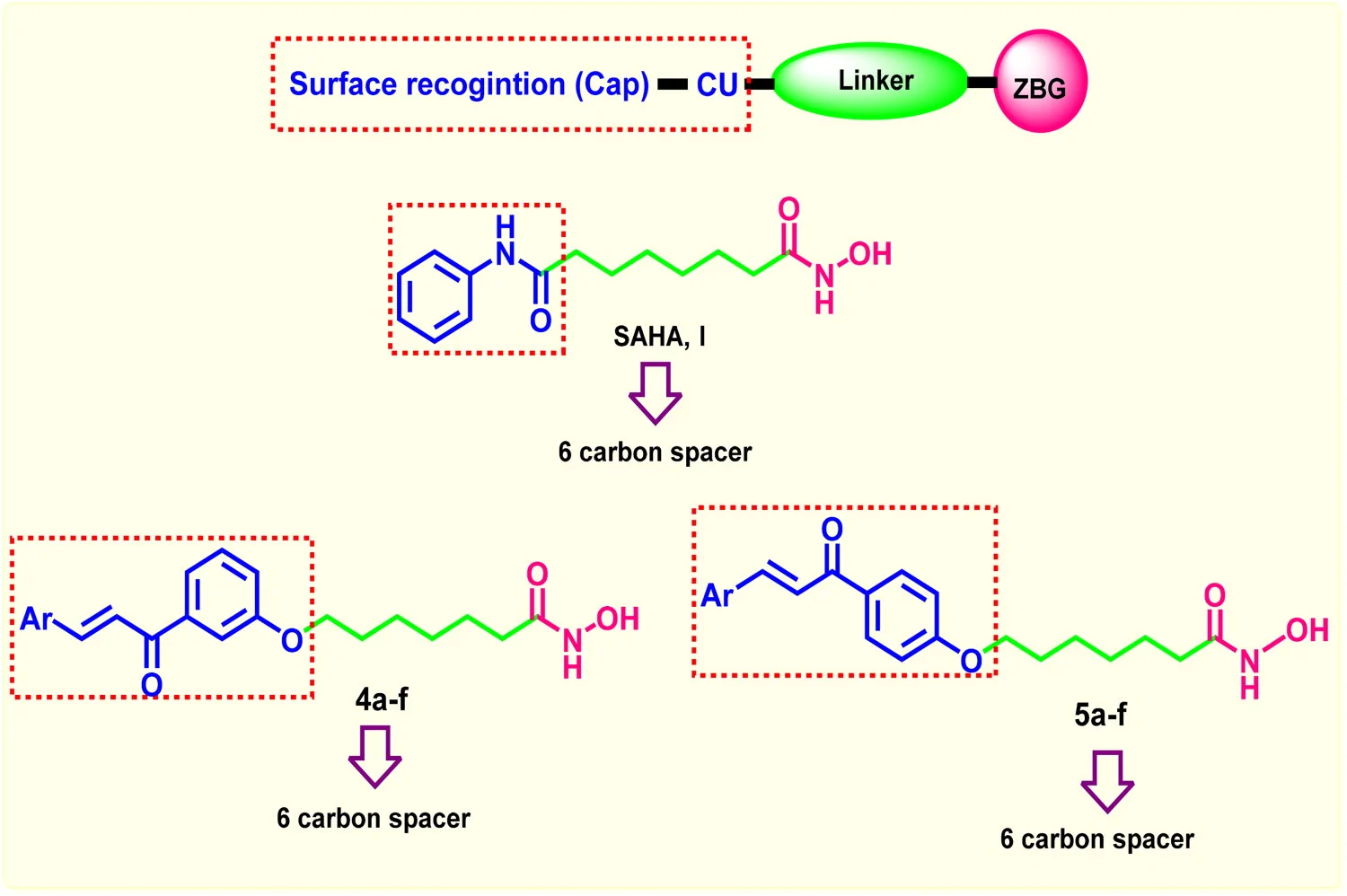
Designed structure of the targeted chalcone hydroxamate **4a-f**, **5a-f** comparable with the reference drug SAHA as HDAC hybrid inhibitors.

In accordance, the current study aims at designing novel HDAC inhibitor hybrids (**4a-f**) and (**5a-f**) comprising the essential mandatory pharmacophores for HDAC inhibition: 1- utilising a chalcone bioactive entity as a cap group in order to improve the efficacy and/or minimise the expected side effects, 2- applying ether oxygen as a connecting unit, 3- retaining of the six-carbon linker conferred by the SAHA reference structure, and 4- keeping the considerable acid hydroxamate ZBG (Figure 4). This study deals with whether that design strategy might be of value in treating disseminating PC causing OS. Therefore, we investigated an *in vitro* cytotoxic effect of the novel synthesised compounds on CRPC cell lines **(Du-145** and **PC-3)**, as well as osteosarcoma **(MG-63)** along with the detection of the cell cycle, apoptosis, and migration, aiming to promote the HDAC enzyme inhibition potency, decrease the side effects, or improve the oral pharmacokinetics that require further investigation.

## Results and discussion

### Chemistry

**Scheme 1.**
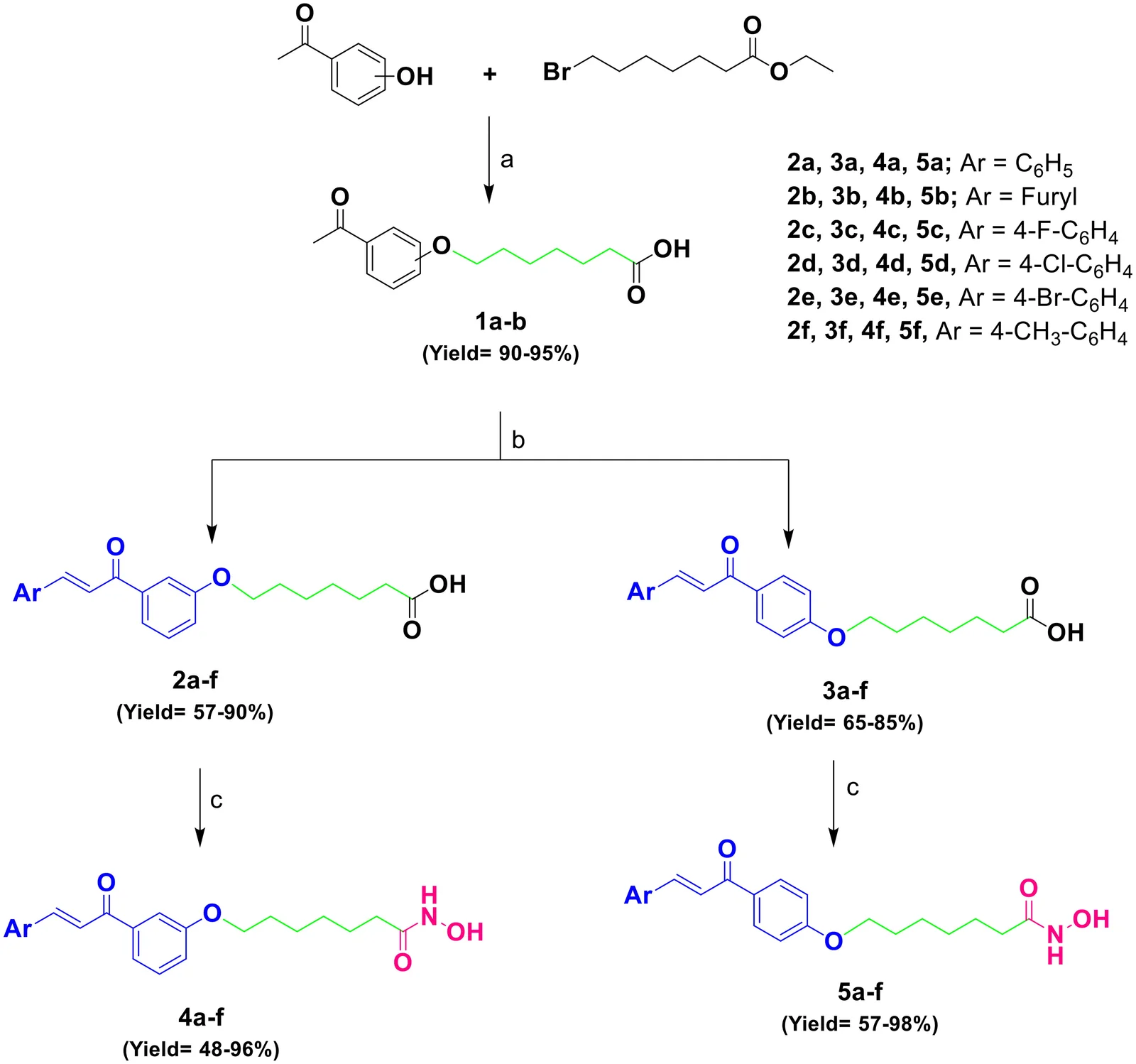
Synthesis of the target derivatives chalcone-HDAC inhibitor hybrids **4a-f** and **5a-f.**

Preparation of the target chalcone/HDAC inhibitor hybrids has been described in Scheme 1. The synthetic strategy starting with the preparation of the two starting acids **1a** and **1b** were *via* dropwise addition of ethyl-7-bromoheptanoate to 3-hydroxyacetophenone or 4-hydroxy acetophenone in the presence of K_2_CO_3_ as a base and stirred at 70–80 °C for 7–8 h^37^^,^ followed by acidic workup of the reaction mixture using diluted HCl to afford the desired compounds **1a** or **1b** (**Scheme 1**) in a good yield; 90 and 95%, respectively. 7–(4-acetylphenoxy) chalcone-carboxylic acid derivatives **2a-f** and **3a-f** were prepared *via* Claisen- condensation reaction^38^ between 7–(3-Acetylphenoxy) heptanoic acid **1a** or 7–(4-Acetylphenoxy) heptanoic acid **1b** and the appropriate aldehyde derivatives in the presence of sodium hydroxide using ethanol as a solvent and stirring at room temperature for 12 h. (Scheme 1). ^1^HNMR of compounds **2a-f** and **3a-f** showed peak for carboxylic acid proton at 12.03–12.32 ppm. Also,^13^ CNMR showed three peaks at 189.3–187.2, 175.3–174.9, and 163.3–159.5 ppm related to (C = O), (COOH), and (Ar-C-O), respectively.

**Reagents and conditions**: (a) i, DMF, K_2_CO_3_, bromo esters, 70–80 °C, 12h reflux, dil. HCl; (b) appropriate aldehyde, NaOH, Ethanol, r.t, 3h, HCl; (c) CDI, ACN, 1–2 h., r.t, NH_2_OH, 3 h.

All the newly prepared derivatives were confirmed by ^1^H-NMR, ^13^C-NMR and elemental analyses. Finally, we adopted a previously reported mild and efficient method for the preparation of hydroxamic acid derivatives **4a-f** and **5a-f** that is suitable for a variety of substituted carboxylic acids^38^. The target compounds **4a-f** and **5a-f** were prepared using a previously reported simple and efficient one-step procedure by converting 3-substituted acryloyl phenoxy heptanoic acid derivatives containing chalcones **2a-f** and **3a-f**, respectively, into their imidazolide derivative by reaction with *N, N’*-carbonyl diimidazole (CDI) in dry acetonitrile at room temperature. This was further reacted with hydroxylamine hydrochloride to afford the corresponding hydroxamic acid-containing derivatives **4a-f** (Scheme 1). The proposed structures of the newly synthesised compounds **4a-f** were confirmed by ^1^H NMR, ^13^C NMR and elemental microanalyses. ^1^H NMR spectra for compounds **4a-f** and **5a-f** showed disappearance of carboxylic acid proton and appearance of new NH peak at 10.43–10.30 ppm. Also, ^13^C NMR of compounds **4a-f** and **5a-f** showed upfield shift of the carboxylic acid carbon from 175.3–174.9 to 163.3–159.5 ppm due to its conversion to (C = O-NH). As an example, ^1^H NMR spectrum of compound (*E*)-7–(3-(3-(Furan-2-*yl*)acryloyl)phenoxy)-*N*-hydroxy heptanamide (**4b**) is characterised by the disappearance of COOH proton and the appearance of a singlet signal in the offset region at δ 10.41 ppm attributed to the -NH proton of the hydroxamic acid group, appearance of another singlet at δ 9.14 ppm corresponding to the -OH of the hydroxamic acid group, one doublet signal at δ 7.69 ppm corresponds to one proton of the chalcone group (CH = CH-C = O) with *J* = 16 Hz, four doublets at δ 7.56, 7.51, 7.47 and 7.15 ppm with *J* = 8 Hz, resemble the four aromatic protons and one singlet signal at δ 6.71 ppm of another aromatic proton. Moreover, two triplet signals at δ 4.06 and δ 2.23 ppm with *J* = 8 Hz, resemble the (O-CH_2_) group and (H_2_C-C = O) group of the aliphatic linker, respectively. The remaining aliphatic and aromatic protons were all detected in their anticipated regions. The ^13^C NMR spectrum of compound **4b** revealed a characteristic peak at δ 187.28 ppm corresponds to C = O group, another peak at δ 169.62 ppm which was assigned for carbonyl carbon of CONHOH group. The peak at δ 163.13 ppm was assigned for the carbon in the aromatic ring attached to the oxygen. Two other peaks which represent the two carbons of the chalcone group were observed at δ 146.34 and δ 130.71 ppm. Moreover, another characteristic peak at 68.34 ppm confirmed the aliphatic O-CH_2_ group next to the oxygen. All other aromatic and aliphatic carbons were detected in their expected regions. The elemental analyses data is consistent with the calculated confirming the suggested structure.

^1^H NMR spectrum of (*E*)-*N*-hydroxy-7–(4-(3-(*p*-tolyl)acryloyl)phenoxy)heptanamide **5f**, as a representative example, is also characterised by the disappearance of COOH proton accompanied by the presence of a singlet signal in the offset region at δ 10.38 ppm attributed to the -NH proton of the hydroxamic acid group and the appearance of another singlet at δ 9.07 ppm corresponding to the OH of the hydroxamic acid group, one doublet signal at δ 7.87 ppm corresponds to one proton of the chalcone group (CH = CH-C = O) with *J* = 16 Hz, another doublet signal at δ 7.67 ppm corresponds to the other proton of the chalcone group (CH = CH-C = O) with *J* constant = 16 Hz, four doublets at δ 8.14, δ 7.76, δ 7.27 and δ 7.07 ppm with *J* = 8, resemble the eight aromatic protons. Moreover, two triplet signals at δ 4.08 and δ 1.97 ppm with *J* = 8, resemble the (O-CH_2_) group and (H_2_C-C = O) group of the aliphatic linker, respectively. The remaining aliphatic and aromatic protons were all detected in their anticipated regions.

The ^13^C NMR spectrum of compound **5f** revealed a characteristic peak at δ 187.28 ppm corresponds to C = O group, another peak at δ 169.79 ppm which was assigned for carbonyl carbon of CONHOH group. The peak at δ 162.73 ppm was assigned for the carbon in the aromatic ring attached to the oxygen. Two other peaks which represent the two carbons of the chalcone group were observed at δ 143.63 and δ 132.58 ppm. Moreover, another characteristic peak at 68.30 ppm confirmed the aliphatic O-CH_2_ group next to the oxygen. All other aromatic and aliphatic carbons were detected in their expected regions. The elemental analyses data are consistent with the calculated confirming the suggested structure.

### Biological evaluation

#### Cytotoxicity assessment of new compounds

Ongoing through the hydroxamic acid hybrids potential anticancer activity and gaining a primary structure-activity relationship, the cytotoxicity of 12 compounds of two series (**4a-f**) and (**5a-f**) were investigated using standard SRB assay on human prostate cancer cell lines (Du145, PC-3), and osteosarcoma cells (MG-63). Each sample was analysed in triplicate. Most compounds inhibited the viability of the three cancer cells in a dose-dependent manner with IC_50_ values less than 50 µM, as shown in Table 1. The results showed that compounds of two series (**4a-f**) and (**5a-f**) exhibited pronounced cytotoxicity in human prostate cancer cell lines and osteosarcoma.

**Table 1.** IC_50_ (μM) values ± SD (*n* = 3) of hydroxamic acid hybrids **4a-f**, **5a-f**, and Doxorubicin in prostate cancer, osteosarcoma, and non-cancerous HSF cell lines after treatment for 72 h.

Compounds	Cytotoxicity in different cell lines (IC_50_ μM ± SD)					Selectivity index (SI)		
	R	DU-145	PC-3	MG-63	HSF	DU-145	PC-3	MG-63
**4a**	H	15.91 ± 2.6	20.19 ± 3.1	14.09 ± 2.2	25.31 ± 1.77	1.59	1.25	1.80
**4b**	Furyl	1.58 ± 0.47	1.39 ± 0.35	3.53 ± 0.36	7.52 ± 0.56	4.76	5.41	2.13
**4c**	4-F	3.27 ± 0.37	4.97 ± 0.79	16.20 ± 1.38	21.99 ± 1.21	6.72	4.42	1.36
**4d**	4-Cl	2.29 ± 0.42	1.72 ± 0.21	14.00 ± 2.6	5.25 ± 0.63	2.29	3.05	0.38
**4e**	4-Br	3.03 ± 0.86	3.60 ± 0.49	12.18 ± 1.98	3.79 ± 0.81	1.25	1.05	0.31
**4f**	4-CH_3_	3.77 ± 0.14	13.20 ± 1.9	21.71 ± 2.1	8.29 ± 1.71	2.20	0.63	0.38
**5a**	H	2.29 ± 0.87	3.07 ± 0.28	11.17 ± 1.68	17.82 ± 1.44	7.78	5.80	1.60
**5b**	Furyl	1.63 ± 0.25	1.24 ± 0.33	6.46 ± 0.94	4.22 ± 0.28	2.59	3.40	0.65
**5c**	4-F	2.99 ± 0.94	2.81 ± 0.71	14.09 ± 1.78	20.33 ± 1.51	6.80	7.23	1.44
**5d**	4-Cl	0.93 ± 0.1	1.05 ± 0.17	2.14 ± 0.45	6.91 ± 1.74	7.43	6.58	3.23
**5e**	4-Br	2.77 ± 0.79	6.06 ± 1.4	17.34 ± 2.44	5.55 ± 1.14	2.00	0.92	0.32
**5f**	4-CH_3_	2.90 ± 0.93	5.85 ± 0.98	27.26 ± 1.36	4.39 ± 0.92	1.51	0.75	0.16
**Doxorubicin**	---	0.9 ± 0.01	1.03 ± 0.02	0.49 ± 0.01	0.39 ± 0.05	0.43	0.38	0.80

The selectivity indexes (SI) were calculated relative to the cancerous cell lines.

Interestingly, compound **5b** displayed marked cytotoxicity against Du145 (1.63 µM), PC-3 (1.24 µM) and MG-63 cells (6.46 µM). Compound **4b** showed comparable cytotoxicity to **5b** against Du-145 (1.58 µM), PC-3 (1.39 µM), and MG-63 cells (3.53 µM). Compound **5d** showed the best growth inhibitory activity against the three cell lines with IC_50_ values of 0.93 µM (Du-145), 1.05 µM (PC-3), and 2.14 µM (MG-63) cells. Therefore, it was selected for further studies.

Most of the tested compounds displayed greater potency against prostate cancer than the osteosarcoma cells. The results proved that all compounds exhibited very strong anticancer activity against Du-145 (advanced stage) prostate cancer with IC_50_ values < 5 µM except, **4a** with IC_50_ = 15.91 µM. Moreover, the tested compounds showed strong anticancer activity against PC-3 (late stage) with IC_50_ values < 10 µM except for **4a** (IC_50_ = 20.19 µM) and **4f** (IC_50_ =13.20 µM). Unlike prostate cancer, osteosarcoma showed less response to most of the tested compounds with IC_50_ values ranging from 11.17–27.26 µ. In fact, the two prostate cancer cell lines (PC-3 and DU-145) were chosen for their aggressiveness, metastatic origin, and androgen- insensitivity^39^. Therefore, the potent cytotoxic activity of compounds against these cell lines, specifically the osteosarcoma cell line, makes them potential treatments for targeting the interphase between prostate cancer and bone metastasis, which remains a challenge in the clinical management of human prostate cancer^40^.

The cancer specificity of the newly developed series was evaluated towards cancerous cell lines DU-145, PC-3, and MG-63, with safety assessed using normal human skin fibroblasts (HSF) cells.

The selectivity index (SI) values are shown in Table 1. For the Du-145 cell line, most compounds exhibited SI values > 2, indicating good selectivity and low toxicity, with compounds 5a and 5d displaying the highest SI values of 7.78 and 7.43, respectively. In the PC3 cell line, SI values were generally lower; compounds 5c and 5d displayed the best selectivity (7.23 and 6.58), while compounds 4f, 5e, and 5f showed SI < 1 (0.63, 0.92, and 0.75), suggesting higher toxicity and poor cancer selectivity. In MG-63 cells, SI values were lower overall, with 5d and 4b having the best values of 3.23 and 2.31, whereas 5f showed the lowest SI (0.16). Doxorubicin showed the lowest safety and selectivity to cancer cells, with SI values of 0.43 (Du145), 0.38 (PC-3), and 0.80 (MG-63), highlighting the drawbacks of chemotherapy in cancer management.

These findings are consistent with previous work, indicating that HDAC inhibitors exert a more pronounced effect on tumour cells than normal cells, as they selectively modulate gene expression and consequently activate multiple anti-tumour pathways^41^.

To better understanding the mechanism of action of the newly synthesised compounds, the most potent four hybrids with the highest IC_50_ **4d**, **4b**, **5d** and **5b** were chosen for determining their inhibitory activity against HDAC2, 4, 6 and 8 isoforms along with SAHA as a reference drug, as illustrated in Table 2.

**Table 2.** HDAC inhibitory activity of the most active chalcone- hydroxamic acid hybrids (IC_50_ µM).

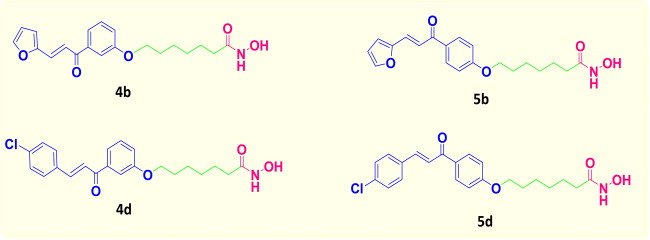				
Hybrid	HDAC 2	HDAC 4	HDAC 6	HDAC 8
**4b**	3.365 ± 0.148	1.782 ± 0.072	2.972 ± 0.12	3.967 ± 0.165
**4d**	0.949 ± 0.042	0.396 ± 0.016	1.295 ± 0.05	2.476 ± 0.103
**5b**	0.572 ± 0.025	0.683 ± 0.027	0.268 ± 0.01	0.881 ± 0.037
**5d**	0.857 ± 0.038	0.165 ± 0.007	1.171 ± 0.045	0.15 ± 0.006
**SAHA (I)**	0.119	4.062	0.019	1.133

#### In vitro HDAC enzymatic inhibitory activity assay

To better understand the mechanism of action of the most potent newly synthesised compounds (hybrids **4d**, **4b**, **5d**, and **5b**) *in vitro* HDAC inhibitory activity was assessed against HDAC2, HDAC4, HDAC6, and HDAC8, with SAHA acting as a positive control.

The HDAC inhibitory activity of the four tested hybrids agrees with the cytotoxic activity. Ongoing through the results, all the tested hybrids **4d**, **4b**, **5d**, and **5b** are more potent than SAHA against HDAC4. as illustrated in Table 2, hybrid **5d** (Ar = 4-chlorophenyl, para-substituted linker) which is the most potent compound on HDAC4, has IC_50_ value of 0.165 µM with 25-fold inhibitory activity than SAHA. Moreover, other hybrids **4d** (Ar = 4-chlorophenyl, meta-substituted linker), **5b** (Ar = 2-furyl, para-substituted linker) and **4b** (Ar = 2-furyl, meta-substituted linker) exhibited 10, 6 and 2-folds inhibitory activity more than SAHA on the same enzyme with IC_50_ values of 0.396, 0.683 and 1.782 µM, respectively.

Regarding HDAC8, hybrid **5d** (IC_50_ value = 0.15 µM) is considered the most potent one, with 8-fold inhibitory activity more than SAHA. Furthermore, hybrid **5b** ranks the second most potent hybrid (IC_50_ = 0.881 µM). Finally, the remaining 2 hybrids, **4d** and **4b**, showed moderate inhibitory activities with IC_50_ value of 2.476 and 3.967 µM, respectively. As a conclusion of the HDAC inhibitory activity, we noticed that SAHA > Para-substituted linker hybrids (**5d** & **5b**) > meta-substituted linker hybrids (**4d** & **4b**) in inhibiting HDAC2 enzymatic activity.

Considering HDAC8 inhibition, Para-substituted linker hybrids (**5d** & **5b**) > SAHA > meta-substituted linker hybrids (**4d** & **4b**), while Para- substituted linker hybrids (**5d** & **5b**) > meta-substituted linker hybrids (**4d** & **4b**) > SAHA on HDAC4 inhibition. On the other hand, SAHA > Para-substituted linker hybrids (**5d** & **5b**) > meta-substituted linker hybrids (**4d** & **4b**) on HDAC6 inhibitory activity. As a result of the previously mentioned data, hydroxamic acid-chalcone hybrids with para-substituted linker are more potent than hydroxamic acid-chalcone hybrids with meta-substituted linker, considering HDAC inhibitory activity assay.

The activity of hydroxamic acid compounds (**4a-f**) and (**5a-f)** was notable in prostate cancer cell lines (Du-145 and PC-3) as well as the osteosarcoma cell line (MG-63), specifically **5d**, which was selected for further assays to investigate its mechanism of action on the three cell lines.

HDACs overexpression is common in various human cancers and is mechanistically linked to tumorigenesis and cancer progression. HDACs regulate apoptosis by dualistic control of epigenetic silencing of apoptosis-related genes and direct post-translational modification of key apoptotic executors. In cell cycle control, HDACs exert spatiotemporal control over gene transcription, DNA replication licencing, and mitotic integrity by modulating the acetylation status of histones and non-histone substrates^42^.

Consequently, HDACs are key therapeutic targets, and HDAC inhibitors (HDAC is) have emerged as promising epigenetic-based cancer therapies, inducing multifaceted antitumor effects, including cell cycle arrest, differentiation, apoptosis, and migration^43^^,^^44^. Therefore, we investigated these assays.

#### Cell cycle distribution of prostate cancer and osteosarcoma cells after treatment with 5d compound

Cell cycle study is crucial to controlling cancer cell proliferation, and cell cycle dysregulation is a hallmark of many malignancies^45^. Therefore, cell cycle distributions in DU-145, PC-3, and MG-63 were analysed using flow cytometry to determine whether the hybrid **5d** disturbs the cell cycle progression in prostate cancer and osteosarcoma.

**Figure 5.**
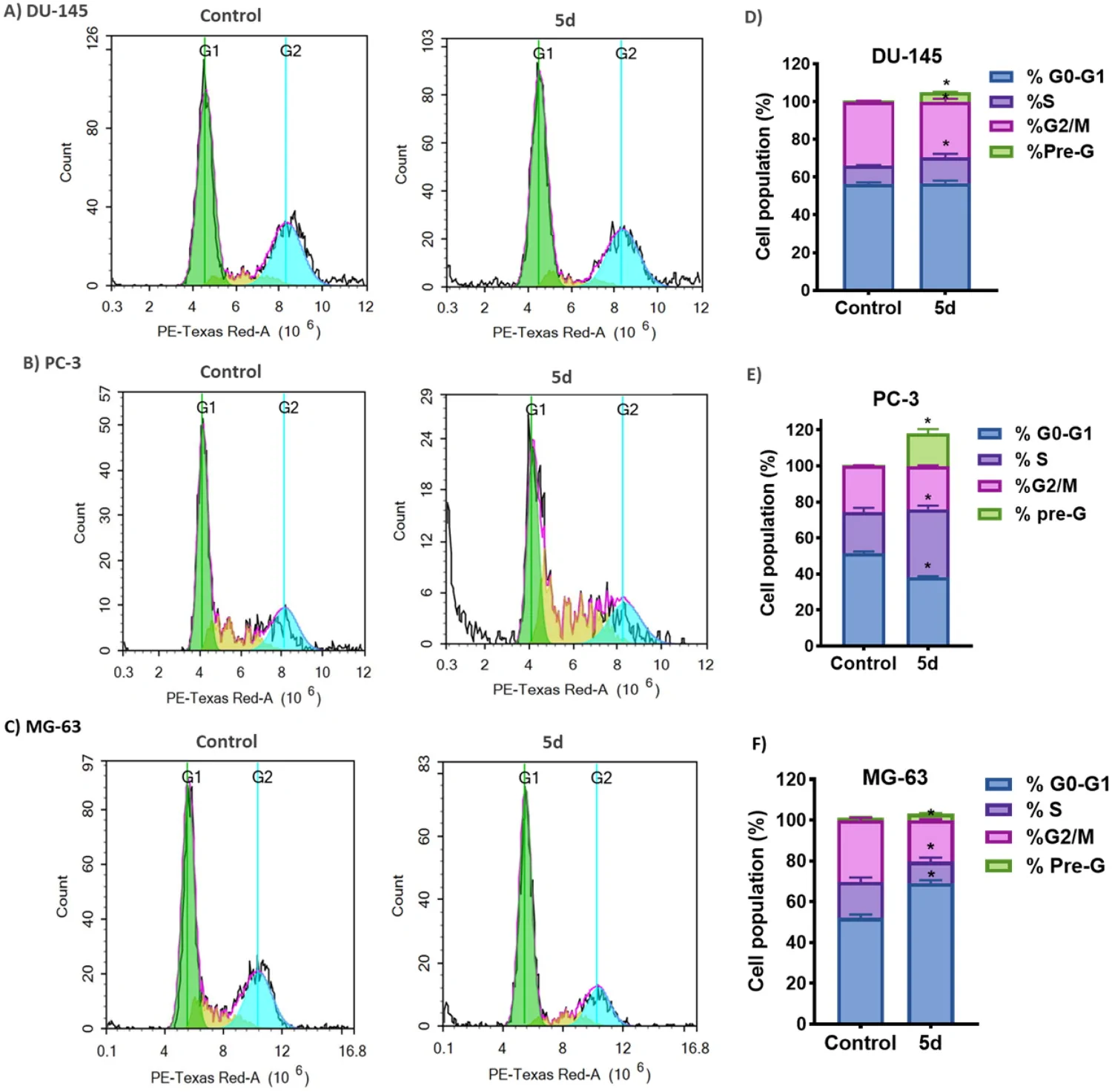
Cell cycle distribution in (A) Du-145, (B) PC-3, and (C) MG- 63 cells after hybrid **5d** treatment for 48 h was determined using DNA cytometry analysis compared with control (untreated cells). **(D), (E) and (F)** Quantification of the percentage of cells in each phase of the cell cycle is illustrated as a bar graph of mean ± SD; *n* = 3. * Statistically significant from control at *p* < 0.05.

Treatment with hybrid **5d** resulted in non-significant changes in all cell cycle phases of DU-145, except pre-G1, which increased from 0.4% to 3.8% compared to untreated control cells (Figure 5(A,D)). However, after hybrid **5d** therapy, the proportion of PC-3 cells in the S phase increased dramatically from 22.80% (control) to 37.63%. By arresting the S phase (DNA replication) of PC-3 cells, hybrid **5d** halted the most critical phase of the cell cycle targeted by many chemotherapeutic drugs, during which genetic information is accurately transmitted to the daughter cells of the M phase division to ensure the stability of genetic traits^46^.

Subsequently, cells cannot enter the mitotic phase, resulting in cell death, as evidenced by a marked increase in the pre-G1 cell population from 1.35% (control) to 21.42%. Reciprocally, we found that the cell population of the G0/G1 phase decreased from 51.59% to 38.34% compared to control cells, while the G2/M phase (mitotic phase) remained unchanged (Figure 5(B,E)).

For osteosarcoma MG-63 cells, hybrid **5d** showed changes in all cell cycle phases, including a significant increase in the cell population of G0/G1-phase (the proportion of proliferating cells) from 54.30% to 71%, demonstrating the ability of hybrid **5d** to inhibit cellular proliferation of osteosarcoma cells through G0/G1 phase arrest^47^. These findings support previous research indicating that HDAC inhibitors trigger G0/G1 cell cycle arrest *via* multiple mechanisms, including the upregulation of cell cycle regulators such as p21 and p53, as well as the downregulation of anti-apoptotic proteins, including Bcl-2^48^.

Furthermore, there was a slight increase in the pre-G1 phase (indicating DNA damage) from 1.35% (control) to 3.10% (hybrid **5d**, treated), along with a decrease in both S phases from 18.20% to 9.6% and G2/M phase from 30.36% to 19.40%, compared with control cells, respectively (Figure 5(C,F)).

The findings revealed that hybrid **5d** has different effects in different phases of the cell cycle of the three cell lines. In contrast to the hybrid **5d** effect in the DU-145 cell line (mainly pre-G1 arrest), it exerted various activity on the S- and the G0/G1-phase of PC-3 and MG-63 cell lines, respectively, demonstrating that different cell cycle responses rely on tumour subtypes^49^. As a result, the current study reveals that hybrid **5d** may have anticancer potential by blocking the cell cycle at various phases and exhibiting a distinct cell cycle arrest pattern. The different observed patterns of cell cycle arrest as a pharmacological endpoint suggest the involvement of multiple mechanisms of action and support the idea that this hybrid **5d** may be a potential therapeutic agent^50^, particularly in metastatic prostate cancer rather than early stage of the disease. These results confirm that the impact of HDAC inhibitors on the cell cycle can vary based on several factors, including the cell line being studied^51^.

#### Hybrid 5d differentially regulates cell death patterns in the prostate and osteosarcoma cells

Cell cycle arrest is intimately associated with apoptosis^52^. Numerous anticancer drugs have been shown to limit cancer cell proliferation and carcinogenesis by inducing cell cycle arrest and apoptosis^53^. Moreover, apoptosis was initially identified as the primary mechanism of HDAC inhibitors-induced cell death^54^.

To determine whether the cytotoxic/antiproliferative effect of hybrid **5d** along with its impact on the cell cycle arrest was due to apoptosis/necrosis, Du-145, PC3, and MG-63 cells for 48 h were stained with PI and Annexin-V/FITC and analysed by flow cytometry.

**Figure 6.**
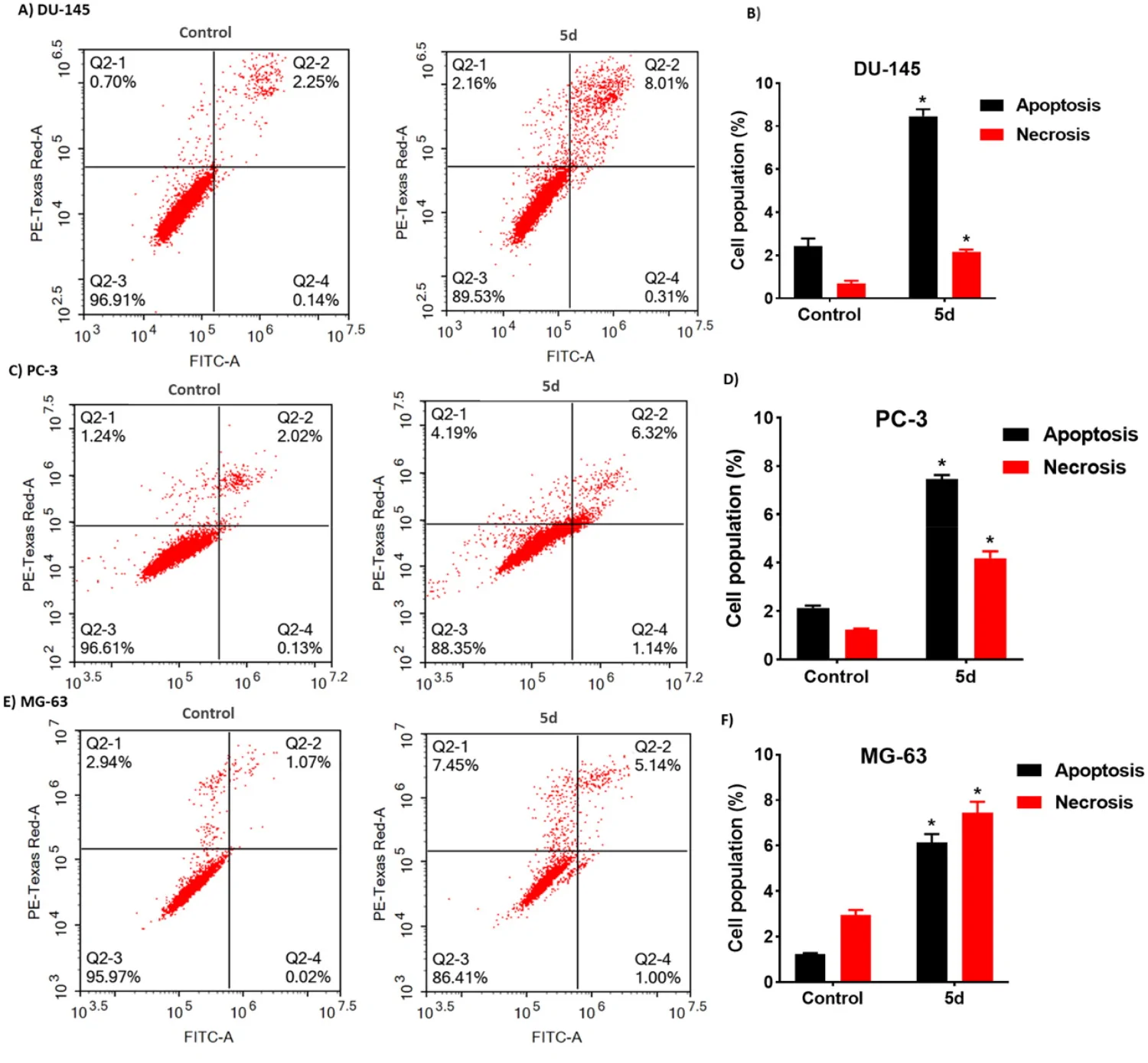
Apoptosis/necrosis assessment in (A) Du-145, (C) PC-3 and (E) MG-63 cells after treatment with hybrid 5d for 48 h. Cells were stained with PI/annexin V-FITC and measured using Flow cytometry. (B), (D) and (F) Different cell populations were plotted as a percentage of total events and presented as mean ± SD; *n* = 3. * Statistically significant from control at *p* < 0.05.

In DU-145 cells, hybrid **5d** caused considerable apoptosis, with an increase from 2.43% to 8.46% compared to control cells and to a lesser amount of necrotic cell death that was nearly 2.16% (Figure 6(A,B)), comparable to 0.7% in control cells. We observed the same scenario in the PC-3 cells, where apoptosis increased from 2.14% to 7.46%, with more necrotic cells around 4.19% compared to 1.24% in untreated cells (Figure 6(C,D)), indicating that prostate cancer relies on apoptosis as a mode of death after hybrid **5d** treatment, specifically that induction of apoptosis is known to be a promising strategy to treat cancer^55^.

In MG-63 cells, the pattern diverges slightly because necrotic cells were moderately dominating, as evidenced by a considerable rise in necrosis from 2.96% to 7.45% and an increase in apoptotic cells from 1.25% to 6.14% as well, with total cell death of 13.59% (Figure 6(E,F)), suggesting a possible application of hybrid **5d** in bone metastatic prostate cancer.

Traditionally, apoptosis is known to be regulated cell death, while necrosis refers to unregulated cell death^56^. Later, results suggested that necrosis is also a form of regulated cell death (also called programmed necrosis or necroptosis)^57^, with the advantage that necrotic signalling pathways were independent of p53-mediated apoptotic signalling pathways since mutated and dysfunctional p53 occurs in more than 50% of cancers^58^. Therefore, hybrid **5d** will be more effective in treating such tumours.

#### Effect of 5d compound on cell migration

Cancer metastasis is depicted by deregulated cell migration and invasion, which poses a substantial obstacle in cancer treatment^59^. Unexpectedly, most available medications are designed to limit tumour growth but are frequently unsuccessful in treating metastatic cancer and identifying an effective therapeutic target to control metastasis in cancer treatment is crucial^60^^,^^61^. Therefore, we performed the migration assay (scratch assay), one of the most extensively used methods for determining the effect of medicines on cell migration and cancer metastasis^61^, to check the potential anti-migratory impact of the hybrid **5d** on the migration of cells.

**Figure 7.**
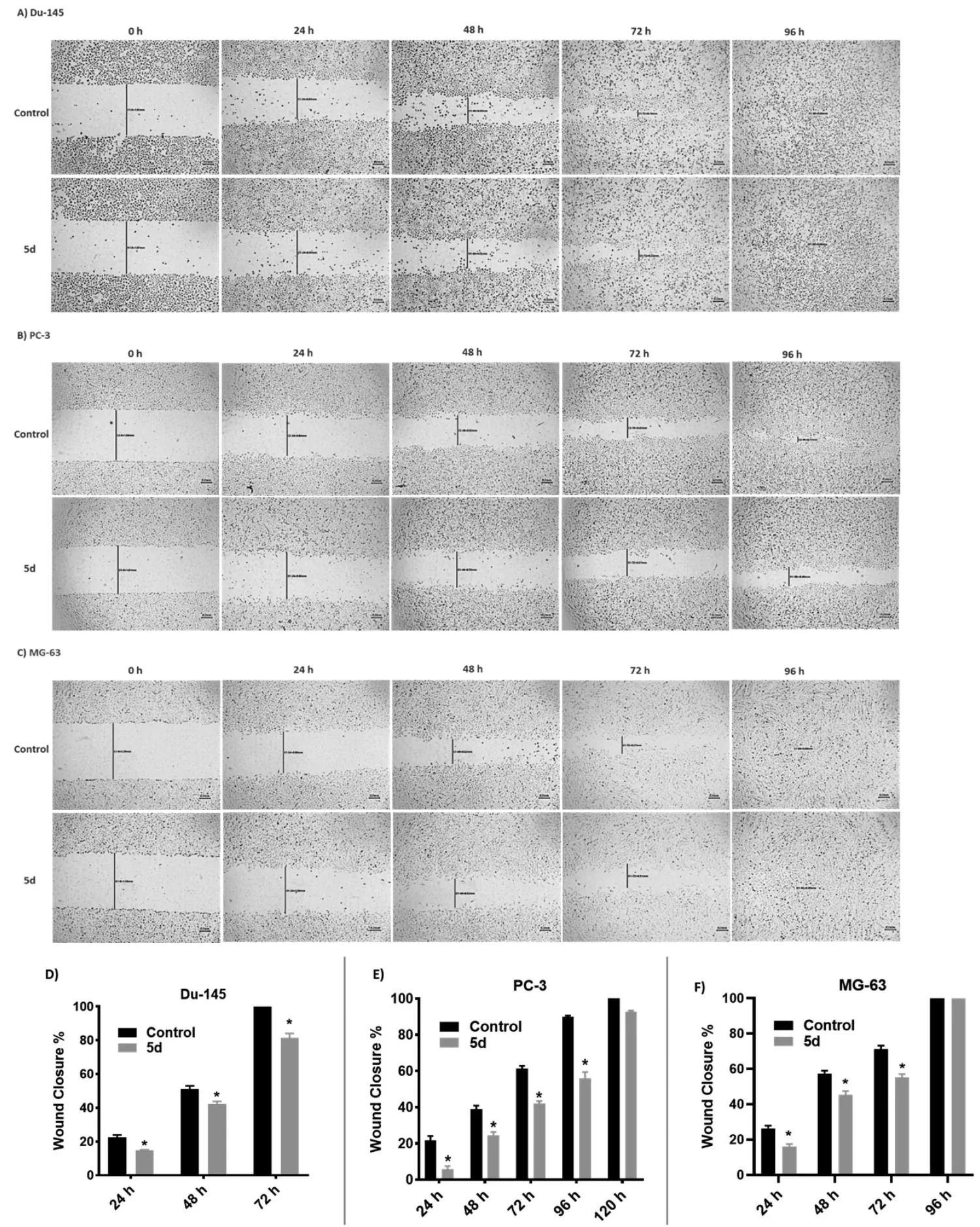
Antimigratory activity of hybrid **5d** on the migration of **(A)** Du-145**, (B)** PC-3 and **(C)** MG-63 cell lines. Migration distances were measured daily for 96 h, except for PC-3, which was monitored for 120 h. The migration distances were measured daily for 72, 120 and 96 h., respectively**. (D), (E)** and **(F)** Data were blotted as wound closure percent % in the three cell lines at each time interval and presented as triplicates. * Statistically significant from control at *p* < 0.05.

Surprisingly, hybrid **5d** demonstrated a potential anti-migratory impact against the three cell lines evaluated. The compound had a significant effect on DU-145 cells (Figure 7(A)). We found that the migration rate of untreated cells was 22.5% and 49.4% at 24 and 48 h., respectively, with 100% closure at 72 h., whereas the migration rate of hybrid **5d**-treated cells was 16.8%, 42.1% and 81.5% at 24, 48, and 72 h., respectively (Figure 7(D)), meaning that the scratch was still open by 18% at 72 h. compared to untreated cells and demonstrated complete scratch closure the following day at 96 h.

Additionally, the compound **5d** was most potent on PC-3 cells (Figure 7(B)), as it decreased the migration rate in the treated cells from 21.7%, 39.2%, 61.3%, 90.7% and 100% at 24, 48, 72, 96 and 120 h. (untreated cells) to 5.9%, 24%, 42.1%, 55.8%, and 92.7% at 24, 48, 72, 96 and 120 h., so it kept the scratch open until the final day (120 h.) (Figure 7(E)), suggesting again that the compound will be an effective treatment in controlling metastasis of aggressive prostate cancer (CRPC) than early-stage disease.

To a lesser extent, MG-63 exhibited the same pattern as DU-145 and PC-3 in terms of delaying cell migration (Figure 7(C)). Untreated control cells migrated at rates of 26.2%, 56.2%, 71.1%, and 100% closure at 24, 48, 72, and 96 h., whereas hybrid **5d**-treated cells migrated at rates of 16.3%, 47.45%, 55%, and 100% at the same time intervals, respectively (Figure 7(F)). Our findings suggested that hybrid **5d** could be an effective treatment for bone metastatic prostate cancer by limiting migratory ability.

These results support previous findings showing that HDAC inhibitors modulate the expression of EMT-associated proteins, thereby suppressing prostate cancer cell migration^62^ and demonstrating anti-migratory effects in osteosarcoma cell lines^63^.

## Molecular docking study

Categorising the HDACs according to their function and phylogenic sequence, class-I HDACs comprise HDAC1,2,3 and 8, while class IIa HDACs involve the HDAC4, 5, 7, and 9. Although the HDAC6 and 10 act as the class IIb HDACs participants^64^. Stringently, the reference hydroxamate containing drug SAHA demonstrated higher selective inhibition of the class I and IIb HDACs subgroups with very lower selectivity to the class IIa isoforms^65^. Molecular modelling analyses were used to investigate the preferred docking positions of the most active compound **5d** within the active sites of the HDAC2, HDAC4, HDAC6, and HDAC6 isozymes. These were carried out alongside Vorinostat (SAHA), the aforementioned reference drug. Using these techniques, we can compare the binding orientation, score, and binding interactions of the investigated **5d** compound to confirm its affinity for these reference ligand-related targets. The docking validation was achieved *via* redocking of the co-crystalized native ligand of each target inside the active site, then RMSD calculation was performed. Interestingly, all RMSD values come compatible with the permissible limit of 2 Å with the following sequence: (0.4987 Å for HDAC2), (0.4062 Å for HDAC4), (1.9746 Å for HDAC6) and (1.9461 Å for HDAC8).

### Molecular docking consideration of compound 5d and the SAHA reference drug inside the HDAC2 active site

Considering the HDAC2 active site, the binding of the reference drug SAHA showed an observed fast rate of association and dissociation drawback, that may limit to some extent its inhibition potential^65^. As our investigated compound **5d** confers zinc chelation in a similar trigonal bipyramidal approach with the reference compound SAHA, with almost identical interacting functional groups positioning. Too, both ligands SAHA and **5d** showed intermediate flexible alkyl chain composed of 6 carbons, that extend through hydrophobic channel like the side chain of a lysine residue. The good news is that our inspected compound **5d** demonstrated higher binding affinity (S= −7.263 Kcal.Mol) than the SAHA ligand (S= −5.668 Kcal.Mol) towards HDAC2, that explains its expected slower association and dissociation rate and hence higher HDAC2 inhibition potential. The binding attitude of the SAHA ligand was translated to the conventional zinc-coordination of the hydroxamate carbonyl group, along with two H-bond donor effects due to both NH groups with Gly154 and Asp104. In addition to that, the H-bond acceptor effect of the hydroxamate carbonyl oxygen with Tyr308 residue. Likewise, the chalcone bearing compound **5d** elucidated similar binding mode with comparable zinc-coordination and H-bonding donor contact with the Gly154 critical residue (2.59Å). Moreover, compound **5d** contacted additionally with 1) the His145 amino acid through H-bond acceptor effect (2.17Å), 2) the Phe155 residue through hydrophobic interaction (5.28Å) due to a phenyl ring of the chalcone core. Besides, the added chalcone radical to compound **5d** was inserted into a hydrophobic foot pocket and surrounded by several hydrophobic residues such as Pro34, Gly32, Tyr29 and Pro106, that may confer its proper fitting and raised binding scoring (Figure 8**: A_1-3_**and **B_1-3_**). The results clarified the higher affinity of the investigated compound **5d** to the HDAC2 active site than the reference ligand SAHA with an expected slower rate of association and dissociation, that could reflect its favourable inhibition.

Figure 8.Shows how compound **5d** and the reference drug SAHA bind to the HDAC-2, 4, 6 and 8 active sites. The native ligand SAHA (**A_1_**, **C_1_**, **E_1_** and **G_1_** panels) and compound under investigation **5d** (**B_1_**, **D_1_**, **F_1_** and **H_1_** panels) are shown in 2D representation inside the HDAC-2, 4, 6 and 8 active sites respectively; in 3D representation as orange sticks, the SAHA ligand (**A_2_**, **C_2_**, **E_2_** and **G_2_** panels) and compound **5d** (**B_2_**, **D_2_**, **F_2_** and **H_2_** panels) co-crystallized with the HDAC-2, 4, 6 and 8 active sites respectively; and in 3D surface depiction as orange space filling, the SAHA ligand (**A_3_**, **C_3_**, **E_3_** and **G_3_**panels) and compound **5d** (**B_3_**, **D_3_**, **F_3_** and **H_3_** panels) at the HDAC-2, 4, 6 and 8 protein surfaces respectively (turquoise space filling).

**Figure:**
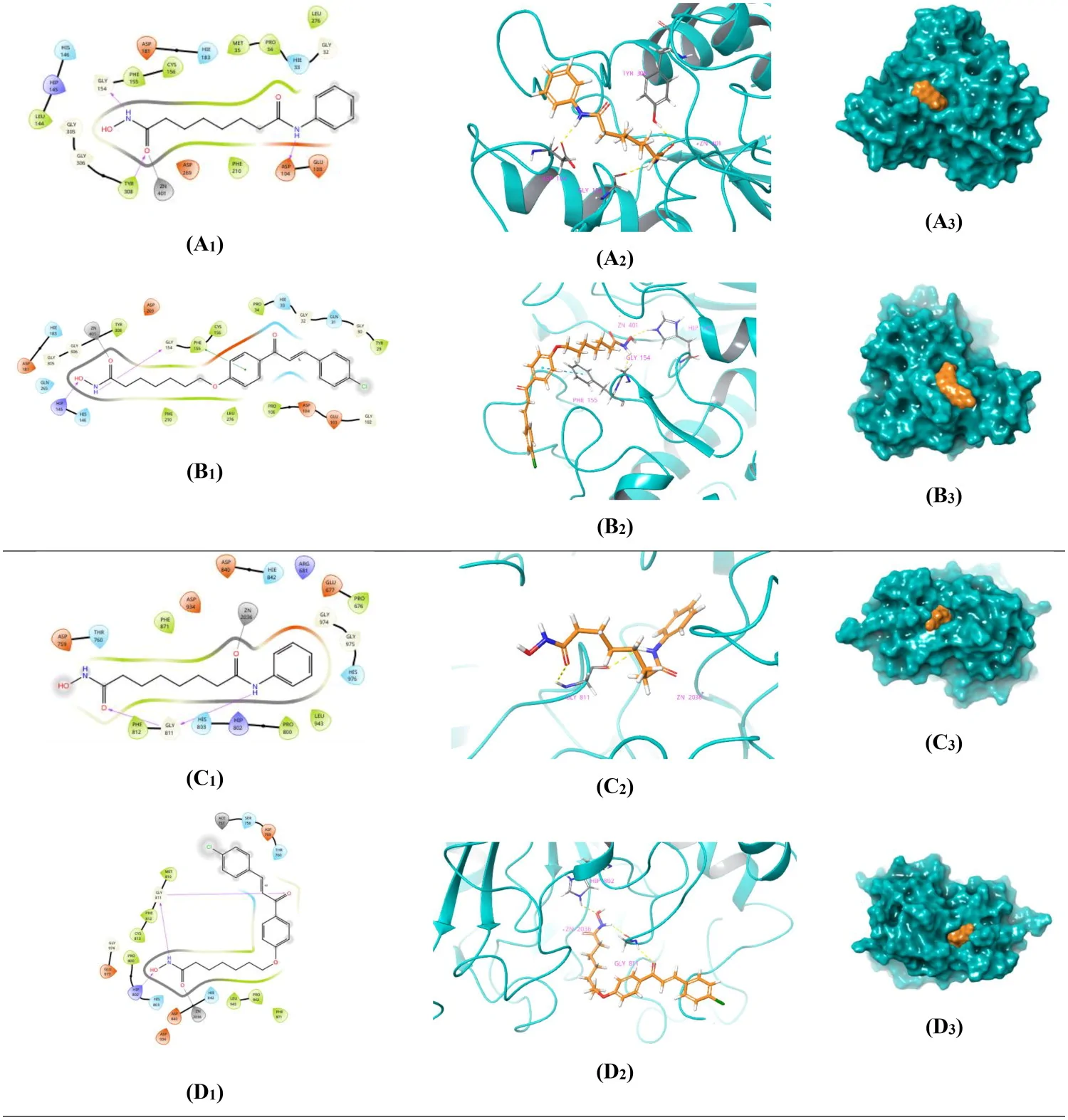

**Figure:**
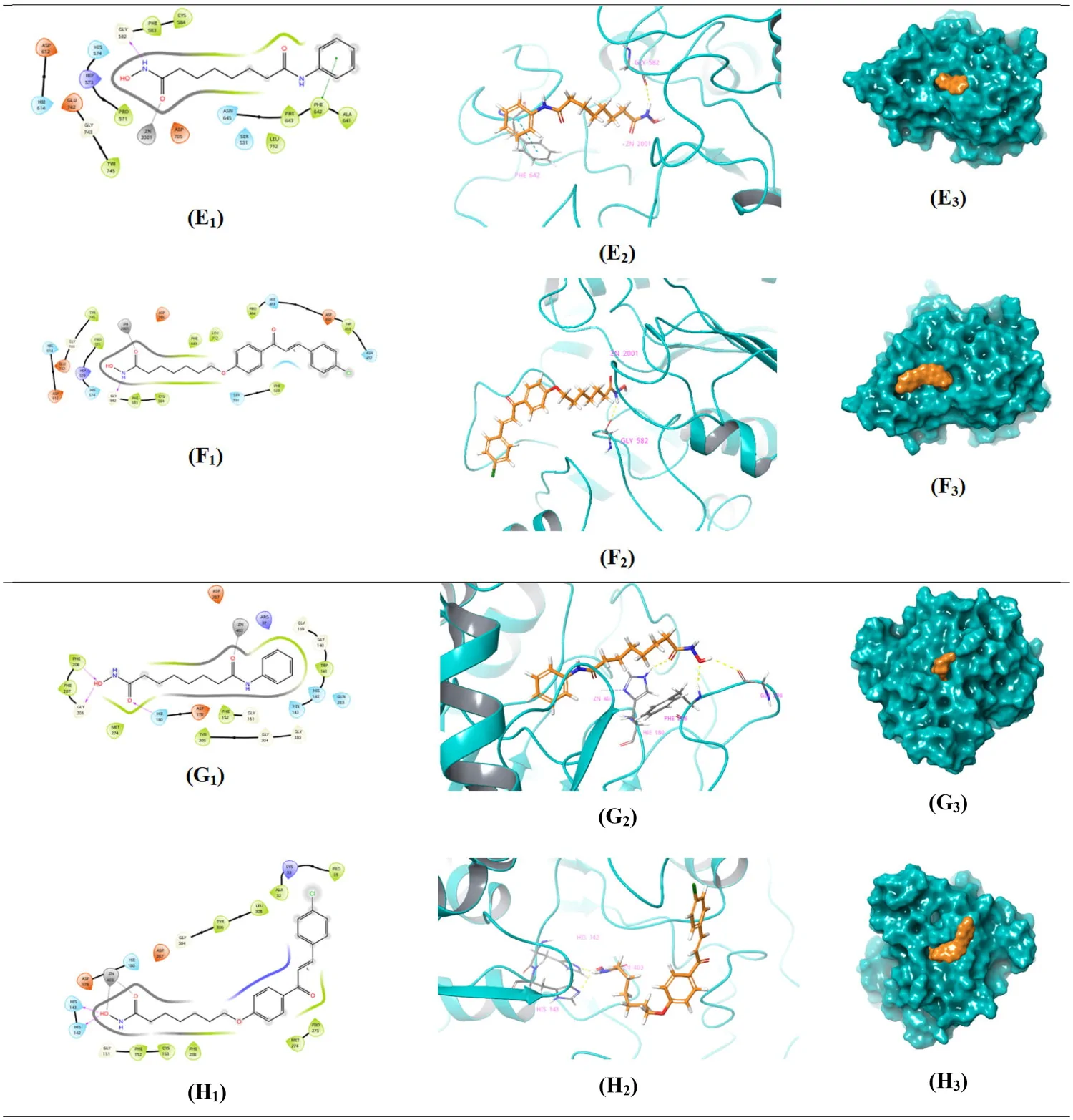

### Molecular docking consideration of compound 5d and the SAHA reference drug inside the HDAC4 active site

Although class IIa HDACs have been suggested as possible therapeutic targets for a variety of cancers, systematic analyses of the function and possible advantages of HDAC-4 inhibition in various malignancies may be found elsewhere^66^. Regretfully, the acid hydroxamate SAHA was considered to be a weak inhibitor to HDAC-4 biotarget with very low selectivity pattern^67^^,^^68^. Herein, after redocking of the SAHA molecule along with the inspected compound **5d** at the HDAC4 active site, the latter proved its higher affinity to HDAC4 active site in a considerable manner with a prominent scoring function of (S = −6.725 Kcal.Mol) that exceeds the SAHA reference control (S = −5.563 Kcal.Mol). Although both SAHA and compound **5d** elucidated zinc-coordination, beside the H-bond donor tendency with the critical Gly811 residue due to the amidic and hydroxamate NH groups, compound **5d** displayed other distinctive H-bond acceptor effect with the His802 residue (2.09Å) due to the hydroxamate hydroxyl oxygen. Also, the proper fitting of the **5d** compound was interpreted into the hydroxamate zinc-coordination instead of the carboxamide zinc-coordination in case of SAHA control (Figure 8**: C_1-3_**and **D_1-3_**). The previous outcomes explained the higher affinity of the chalcone bearing compound **5d** on HDAC4 activity site relative to the SAHA reference, with purposed higher antineoplastic potential that require further study.

### Molecular docking consideration of compound 5d and the SAHA reference drug inside the HDAC6 active site

The HDAC6 is a distinct member of class IIb HDAC isozymes that exhibit great cellular localisation and biological function^69^. The HDAC6 confers unique binding pockets that contain tandem catalytic domains called CD1 and CD2^70^. Focusing on the CD2 cocrystal structure, the catalytic binding of the upcoming substrate *N*-acetyl lysine at 1.82 Å resolution was achieved by forming pentacoordinate zinc ion poised that is necessary for catalysis. On the way for coordination, the *N*-acetyl lysine substrate was fixed by 2 crucial H-bonding with the Ser531 amino acid side chain hydroxyl group, and the Gly582 backbone carbonyl group. Considering the SAHA drug HDAC6 selectivity, that was confirmed through it redocking inside the CD2 active site. It revealed critical H-bond donor effect due to its hydroxamate NH group with the key Gly582 residue, along with its zinc-coordination and an additional π-π* stacking with Phe642 residue. Providently, the investigated compound **5d** confers identical binding attitude inside the CD2 active site of the HDAC6, with identical functional groups coordination with zinc metal and the key Gly582 (2.22Å), beside its higher propagation through a hydrophobic pocket due to its chalcone moiety surrounded by the hydrophobic residues Pro464, His463, Trp459 and Phe533. Nevertheless, compound **5d** achieved Schrodinger binding scoring of −7.524 kcal.Mol, that surpassed the SAHA binding scoring (−5.050 Kcal.Mol) (Figure 8**: E_1-3_**and **F_1-3_**). The results explained the higher selectivity of compound **5d** to HDAC2 CD2 active site, with its favourable binding behaviour with the catalytic Gly582, that hinders the *N*-acetyl lysine substrate binding and hence hinder the HDAC6 catalytic action.

### Molecular docking consideration of compound 5d and the SAHA reference drug inside the HDAC8 active site

Since the class I specific HDAC inhibitors give distinct therapeutic advantage compared to the standard broad-spectrum HDAC inhibitors^71^. Increasing the specificity of a small molecule to the HDAC8 isozyme as a prominent member of class I HDACs, will potentially reveal insights to achieve elevated antineoplastic levels^72^. It was reported that, to increase the HDAC8 selectivity, the inserted molecule should be capable of making zinc chelation with deep reaching into the acetate release channel within the enzyme^73^. The cocrystal structure of the SAHA reference drug after redocking analysis (S= −5.410 Kcal.Mol), reveals its obvious zinc coordination, beside 3 characteristic H-bonding interactions with: 1) His180 (2.15Å), 2) Gly206 (2.64Å), and 3) Phe208 (2.10Å), that exist at the *N*-acetyl lysine substrate tunnel. Remarkably, the investigated compound **5d** achieved an apparently elevated binding score to HDAC8 of −7.059 Kcal.Mol *via* Schrodinger calculation, that is attributed to a perfect zinc chelation *via* the hydroxamate moiety, along with 2 characteristic H-bonding donor effects with His142 (2.04Å), and His143 (2.37Å) (Figure 8**: G_1-3_**and **H_1-3_**) Remarkably, the critical residue His142 serves a dual function in zinc chelation and as a prominent component of the acetate release channel^73^. Hence, the novel compound **5d** accomplished high affinity to the HDAC8 through higher binding scoring and metal chelation, and high selectivity through anchoring inside the acetate release channel that requires further biological assessment.

## Molecular dynamic simulation

To explore the dynamic behaviour of the HDAC biotargets in complex with the compound **5d**, as well as the interacting ligand SAHA, we conducted a 100 ns MD simulation.

### MD Simulation of compound 5d co-crystalised with HDAC2 binding site related to the reference drug SAHA

**Figure 9.**
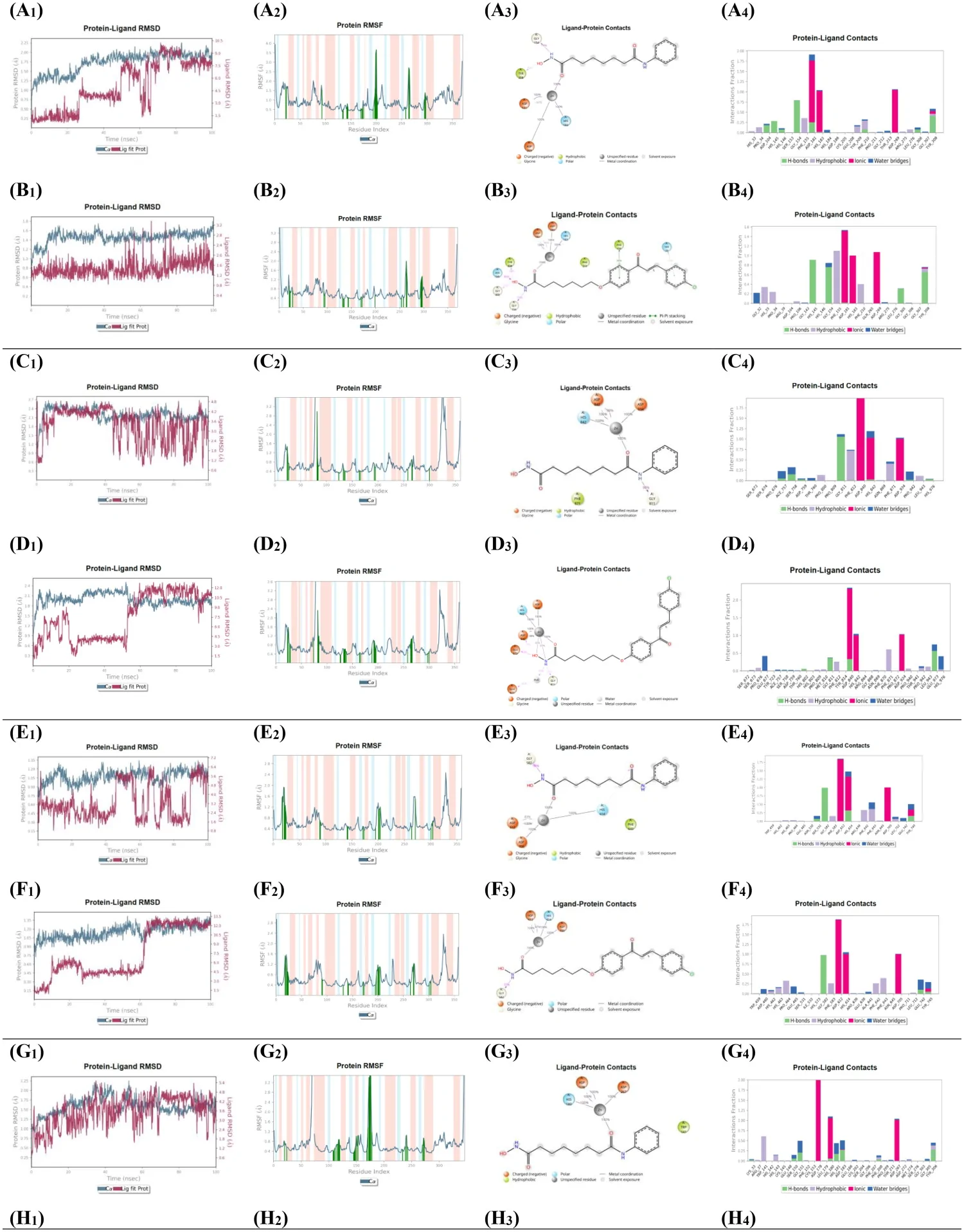
MD simulation results of the inspected compound **5d** comparable with the reference drug SAHA complexed with the active sites of HDAC-2, 4, 6 and 8 proteins. Protein and ligand RMSD illustrated by **(A_1_, C_1_, E_1_** and **G_1_** panels for SAHA**)** and **(B_1_, D_1_, F_1_** and **H_1_** panels for **5d)**, where the HDAC proteins are shown in gray while the **5d** and SAHA ligands are in red, **(A_2_, C_2_, E_2_** and **G_2_** panels for SAHA**)** and **(B_2_, D_2_, F_2_** and **H_2_** panels for **5d)** refer to the HDAC proteins RMSF (root main square fluctuation), **(A_3_, C_3_, E_3_** and **G_3_** panels for SAHA**)** and **(B_3_, D_3_, F_3_** and **H_3_** panels for **5d)** demonstrate the 2D display of ligands-protein contacts, and **(A_4_, C_4_, E_4_** and **G_4_** panels for SAHA**)** along with **(B_4_, D_4_, F_4_** and **H_4_** panels for **5d)** denote the histogram illustrating ligand-protein contact.

The MD simulation was conducted for 100 ns in a solvated environment to assess the dynamic behaviour of the HDAC2 binding domain in complex with the reference drug SAHA and the investigated chalcone compound **5d**. Additionally, we evaluated the sturdiness of the formed complexes between the selected ligands and the HDAC2 active site. Regarding the protein RMSD analysis of the HDAC2 complexes with SAHA and compound **5d**, the Cα atom fluctuations remained within approximately 2.25 Å for SAHA and 1.80 Å for compound **5d** (both starting from 1.0 Å) throughout the simulation, that respond the permissible RMSD fluctuation limit of 3.0 Å. Furthermore, the ligand RMSD, after initial fluctuations due to equilibration, exhibited distinct patterns: 1) For SAHA, the RMSD increased from 1.0 to 3.0 Å within the first 20 ns, fluctuated between 2.8 and 6.0 Å until 55 ns, and then showed higher fluctuations from 1.50 to 9.0 Å within the first 65 ns, before stabilising between 5.80 and 10.30 Å with a lower fluctuation rate until the end of the simulation. 2) In contrast for compound **5d**, the fluctuation rate remained more stable, ranging from 1.0 Å to 3.4 Å throughout the simulation (Figure 9**: A_1_, B_1_**). The protein RMSF pattern of the formed complexes highlighted α-helices and β-strands with red and blue backgrounds, respectively, while loop regions were marked in white. Additionally, vertical green lines on the X-axis of the plot indicated the residues in the protein active site that interacted with the ligands. Notably, both SAHA and compound **5d** interacted with 15 key residues within the HDAC2 binding domain: His33, Pro34, Asp105, His145, His146, Gly154, Phe155, Asp181, His183, Phe210, Asp269, Arg275, Leu276, Gly306, and Tyr308. This confirmed that compound **5d** followed a similar binding pattern to SAHA within the HDAC2 active site. However, compound **5d** exhibited additional interactions with seven extra residues: Gly32, Arg39, Pro106, Gly143, Gln265, Gly305, and Gly307 (Figure 9**: A_2_, B_2_**). The ligand-protein contact map, along with the 2D representation and histogram, was observed in panels **A_3_** and **A_4_**(for SAHA) and **B_3_** and **B_4_** (for **5d**). Importantly, both SAHA and compound **5d** participated in zinc coordination through Asp181, His183, and Asp269, with an interaction fraction of nearly 100%. SAHA formed two characteristic hydrogen bonds with Gly154 (79%) and Tyr308 (30%). Meanwhile, the **5d**-HDAC2 complex exhibited significant π-π* stacking interactions with Phe155 (89%) and His33 (33%), aligning with docking results. These findings suggest that compound **5d** forms a stable and reliable complex with the HDAC2 binding pocket, demonstrating a binding pattern similar to the reference standard SAHA. This supports its potential as a predicted HDAC2 inhibitor.

### MD Simulation of compound 5d co-crystalised with HDAC4 binding site related to the reference drug SAHA

The RMSD analysis of the HDAC4 protein complexed with the native ligand SAHA and the investigated compound **5d** revealed stable fluctuations of the Cα atoms, reaching up to 2.70 Å for SAHA and 2.50 Å for compound **5d**, both starting from 1.50 Å. This confirmed the stability of the **5d**-HDAC4 complex, as its protein RMSD remained within the acceptable limit. Regarding ligand RMSD equilibrium, the SAHA ligand exhibited fluctuations reaching 4.80 Å, starting from 0.80 Å, with high variability from 45 ns until the end of the simulation. In contrast, compound **5d** showed fluctuations ranging from 1.50 Å to 9.0 Å during the first 60 ns, followed by a more convenient range of 6.50 Å to 12.50 Å until the simulation’s completion (Figure 9**: C_1_, D_1_**). The protein RMSF analysis demonstrated that both SAHA and compound **5d** interacted with 16 common residues in the HDAC4 active site: Ser673, Pro676, Ser758, Asp759, Thr760, Pro809, Gly811, Phe812, Asp840, His842, Asn869, Phe871, Asp934, Pro942, Leu943, and His976. This highlighted their similar binding patterns within the HDAC4 pocket. Additionally, compound **5d** exhibited further interactions with 14 additional residues: Ser672, Glu677, Tyr723, His802, His803, Met810, Tyr814, Arg864, Gly868, Phe870, Pro872, Pro940, Thr941, and Glu973, suggesting its potentially higher binding affinity towards the HDAC4 biotarget (Figure 9**: C_2_, D_2_**). The ligand-protein contact histogram revealed that both SAHA and compound **5d** followed a zinc-coordination pattern, interacting with Asp840, His842, and Asp934 with an interaction fraction of 100%. SAHA also formed a strong hydrogen bond with Gly811 (100%), while compound **5d** established hydrogen bonds with Asp840 (33%) and Glu973 (53%), along with a 33% interaction fraction with Gly811. Additionally, compound **5d** exhibited significant water-bridged hydrogen bonding with Glu677 (41%) (Figure 9**: C_3,4_** and **D_3,4_**). These results confirm a superior binding aptitude of the promising chalcone hydroxamate compound **5d** over the SAHA reference drug, further supporting the stability of the HDAC4-**5d** complex throughout the simulation.

### MD Simulation of compound 5d co-crystalised with HDAC6 binding site related to the reference drug SAHA

Fortunately, the RMSD analysis of the HDAC6 protein complexed with the native ligand SAHA and the inspected compound **5d** proved stable fluctuations of the Cα atoms, reaching up to 1.35 Å for SAHA and 1.40 Å for compound **5d** (both starting from 0.75 Å), that remained within the acceptable range. Furthermore, the ligand RMSD equilibrium referred to that the SAHA ligand exhibited fluctuations peaking at 7.0 Å, starting from 0.8 Å, with a high fluctuation rate. In contrast, compound **5d** fluctuated between 1.50 Å and 6.0 Å during the first 60 ns, then stabilised within the range of 10.5 Å to 13.50 Å until simulation ending (Figure 9**: E_1_, F_1_**). Regarding the protein RMSF analysis, both SAHA and compound **5d** interacted with 17 common residues in the HDAC6 active site, that were: Trp459, His462, His463, Pro464, Glu465, Ser531, Gly582, Phe583, Asp612, His614, Arg636, Phe642, Asn645, Asp705, Leu712, Glu742, and Tyr745. These similar interactions emphasise their comparable binding patterns within the HDAC6 pocket (Figure 9**: E_2_, F_2_**). The histogram of ligand-protein contact revealed that both SAHA and compound **5d** adopted an identical zinc-coordination pattern, interacting with Asp612, His814, and Asp705, each with an interaction fraction of 100%. Additionally, both ligands formed strong hydrogen bonds with Gly582, with interaction fractions of 98% for SAHA and 97% for compound **5d**. Moreover, SAHA displayed notable hydrogen bonding with His614 (31%) (Figure 9**: E_3,4_** and **F_3,4_**). Overall, these findings confirm the stability of the HDAC6-**5d** complex throughout 100 ns simulation, along with the ability of compound **5d** to elucidate a binding mode similar to the reference drug SAHA.

### MD Simulation of compound 5d co-crystalised with HDAC8 binding site related to the reference drug SAHA

The RMSD analysis of the HDAC8 active site complexed with the reference ligand SAHA and the chalcone compound **5d** revealed consistent fluctuations in the Cα atoms, reaching up to 2.25 Å for SAHA and 1.70 Å for compound **5d**, both starting from 1.0 Å throughout the simulation. Regarding ligand RMSD equilibrium, SAHA exhibited fluctuations peaking at 5.40 Å, starting from 1.20 Å. In comparison, compound **5d** displayed fluctuations within a range of 8.0 Å to 15.0 Å, indicating some conformational variations **(**Figure 9**: G_1_, H_1_)**. Besides, the protein RMSF analysis showed that both SAHA and compound **5d** interacted with 22 common amino acids in the HDAC8 active site: Lys33, Trp141, His142, His143, Lys145, Glu148, Ser150, Gly151, Phe152, Asp178, His180, His181, Lys202, Gly206, Phe207, Phe208, Pro209, Asp267, Met274, Gly303, Gly305, and Tyr306 **(**Figure 9**: G_2_, H_2_)**. This confirms their similar binding patterns within the HDAC8 active site between both the investigated ligands. The ligand-protein contact histogram demonstrated that both SAHA and compound **5d** chelated the zinc ion through three key residues Asp178, His180, and Asp267 each with an interaction fraction of 100%. Remarkably, in alignment with docking calculations, compound **5d** formed H-bonds with His142 (66%) and His143 (85%). As well, it exhibited hydrophobic interaction with Phe152 (33%) **(**Figure 9**: G_3,4_** and **H_3,4_)**. These results clarified that compound **5d** exhibits binding similarity to the reference ligand SAHA, supporting their shared mechanism of action.

## Prediction ADME parameters

**Figure 10.**
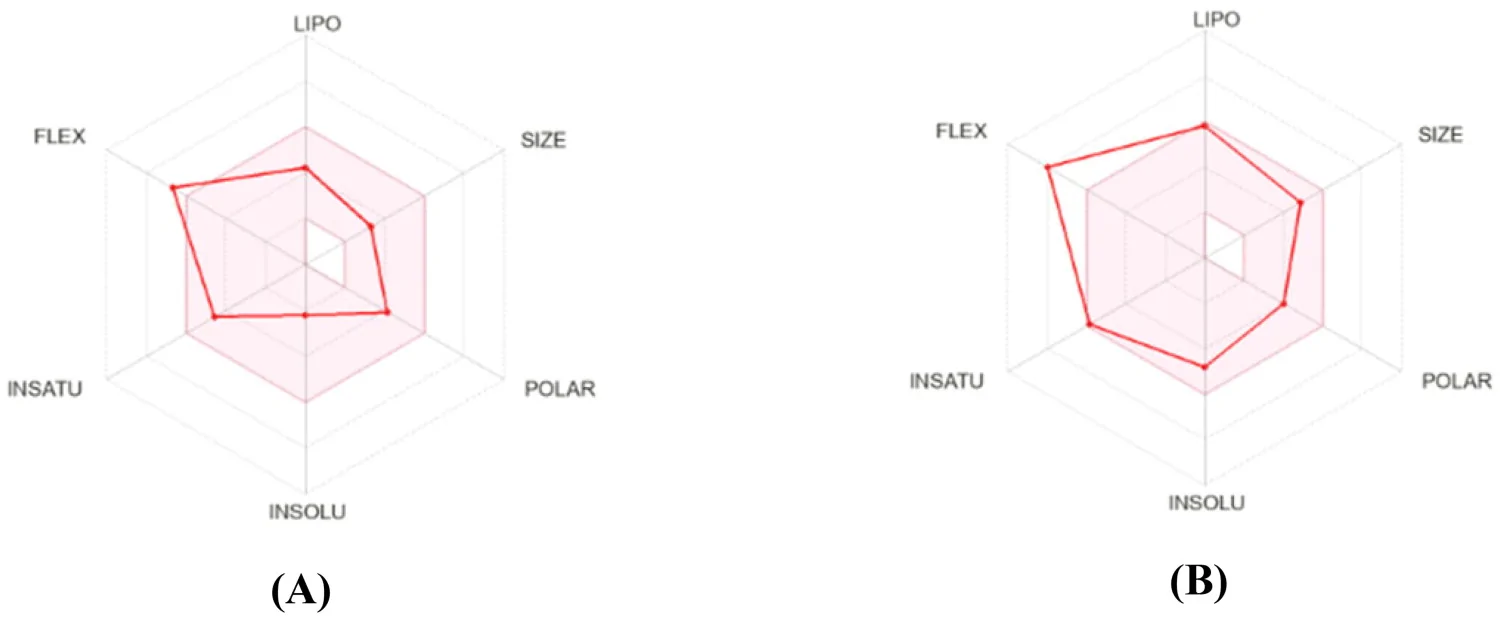
Oral bioavailability representation diagram, compare the oral aptitude of the reference drug SAHA (A) against the investigated compound **5d** (B). The red zone denotes the drug size, lipophilicity, polarity, insolubility, insaturation and flexibility.

The anticipated physicochemical considerations were calculated using the online program SwissADME. The attained results give a positive idea about feasibility profiles of the novel synthesised candidate **5d** related to the reference drug SAHA. Providently, compound **5d** exhibited two hydrogen bond donors (HBD) and four hydrogen bond acceptors (HBA), which fall within the acceptable limits defined by Lipinski’s rule of five, no more than five HBDs and ten HBAs. Considering the lipophilicity parameter, the atomistic knowledge-based method (XLOGP3) must be less than 5.00 to achieve the adequate lipophilic attitude needed for oral absorption^74^, while compound **5d** achieved XLOGP3 permissible limit of 4.96 that exceeds the SAHA reference (1.86). Also, in a provident manner both **5d** compound and SAHA showed lower molecular weight than the required limit for oral administration (500)^75^ that explains their suitable molecular size, while the inspected compound **5d** represented higher molecular weight than SAHA within the acceptable limit (Table 3). Regarding the drug polarity, the oral administrated compound should reveal topological polar surface area (TPSA) less than 130Å^2,^^76^ that support our novel compound **5d** (TPSA= 75.63 Å^2^) related to SAHA (TPSA= 78.43 Å^2^). In addition, the water solubility parameter (insolubility), represented by Log S (ESOL) that must be lower than 0 value for oral acceptance, was suitably achieved in both **5d** and SAHA that exhibited Log S (ESOL) values of −4.79 and −2.22 respectively, whereas the **5d** compound exerted lower water solubility. Too, both **5d** compound and SAHA displayed fraction Csp3 value of 0.27 and 0.43 respectively, that are less than 1.00 and so they exhibit a permitted insaturation level^77^. Unfortunately, both compounds **5d** and SAHA exhibited 12 and 10 rotatable bonds, respectively, exceeding the recommended threshold for optimal oral bioavailability and potentially representing a pharmacokinetic limitation. However, the increased flexibility of the linker region may favour accommodation within the HDAC binding channel and enhance target interactions that require further biological evaluation (Figure 10). Lastly, both **5d** and SAHA compounds complied with Lipinski’s rule of five, indicating favourable drug-likeness characteristics. Compound **5d** exhibited higher lipophilicity (XLOGP3 = 4.96) and lower aqueous solubility than SAHA while remaining within the acceptable physicochemical range for orally active compounds. However, these properties should be interpreted cautiously, as increased lipophilicity may improve membrane permeability but may also adversely affect aqueous solubility and pharmacokinetic behaviour. Therefore, the overall oral bioavailability of compound **5d** cannot be inferred solely from *in silico* physicochemical parameters and requires further experimental evaluation.

**Table 3.** Predicted ADME parameters of the chalcone hydroxamate compound **5d** against SAHA.

Comp.	HBD	HBA	Lipophilicity	Size	Polarity	Insolubility	Insaturation	Flexibility	Druglikeness
			XLOGP3	MWt	TPSA(Å^2^)	Log S (ESOL)	Fraction Csp3	Num. rotatable bonds	Lipinski
**5d**	2	4	4.96	401.88	75.63	−4.79	0.27	12	Yes
**SAHA**	3	3	1.86	264.32	78.43	−2.22	0.43	10	Yes

## Structure–activity relationship

Based on the biological activities, the simple structure-activity relationship (SAR) could be summarised as follows: The chalcone aromatic cap group displayed prominent HDAC inhibition potential through the formation of many hydrophobic interactions and hydrogen bonding contacts with critical residues at HDAC active sites according to the docking study. Also, utilising the hexamethylene intermediate chain comparable with the SAHA reference accomplished the optimum distance that allow the acid hydroxamate moiety to confer proper zinc-binding inside the HDAC active sites.

**Figure 11.**
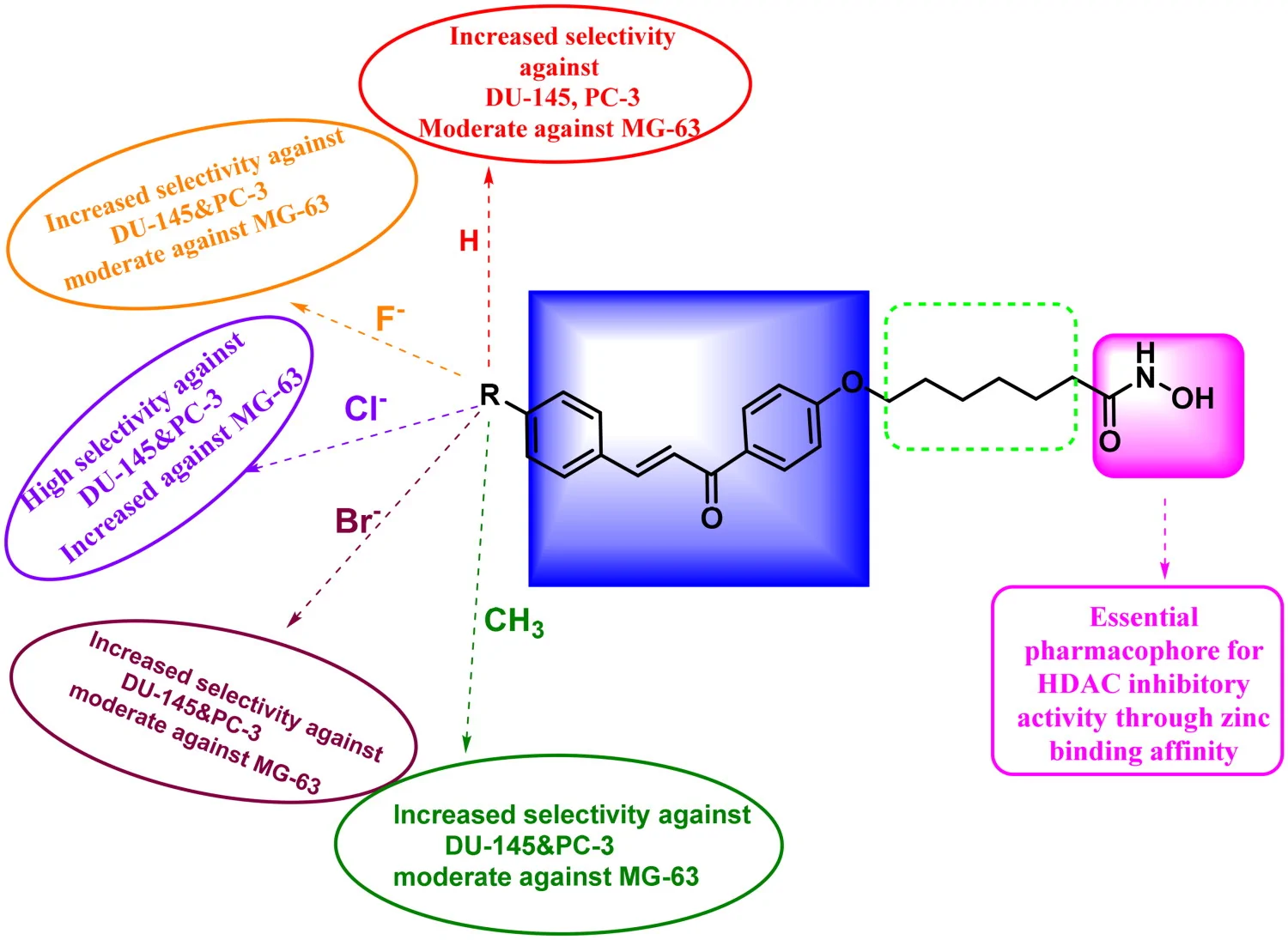
Structure–activity relationship (SAR) for the newly synthesised chalcone/HDACI hybrids **(5a-f)** with para-aromatic cap and hydroxamic acid as ZBG.

**Figure 12.**
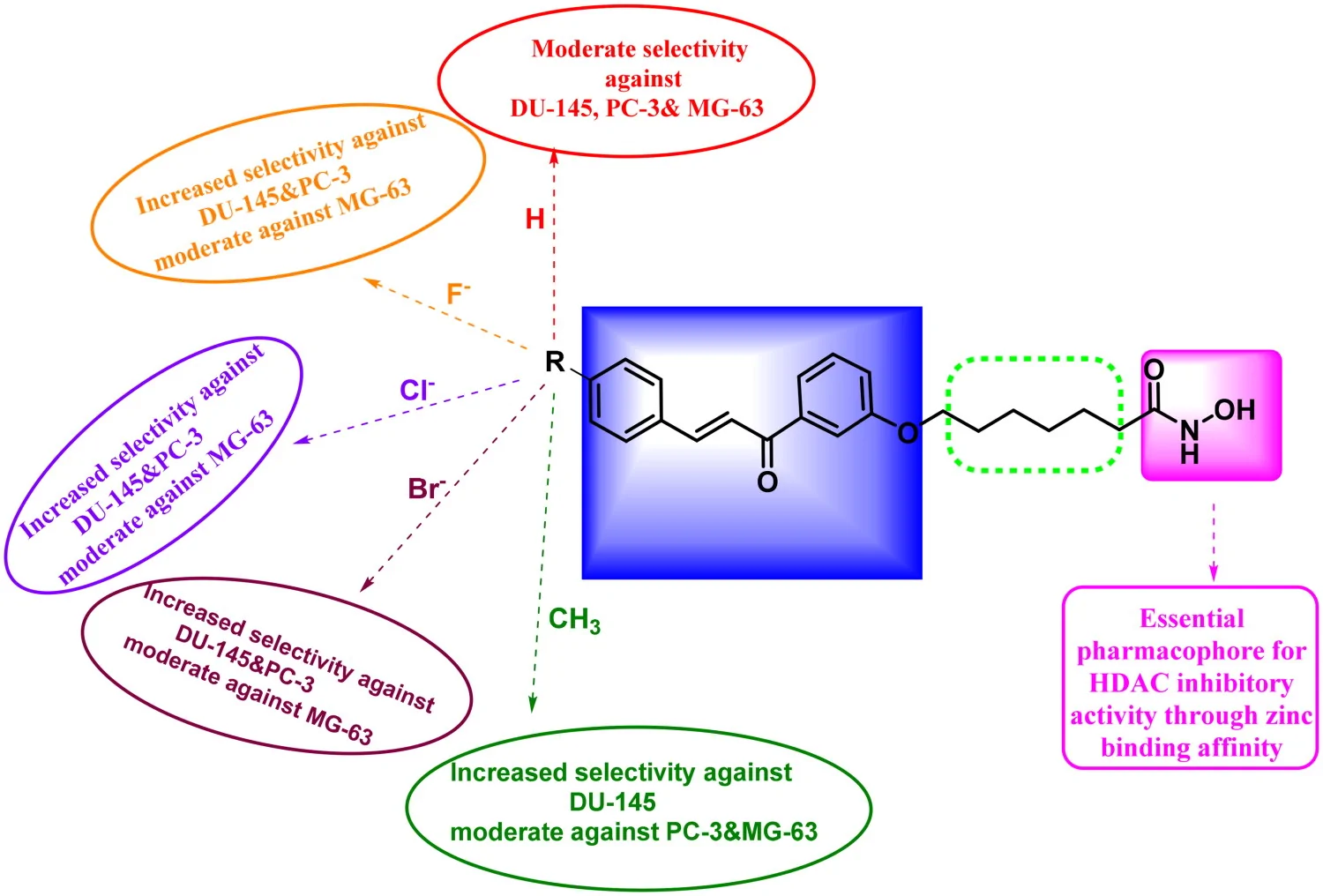
Structure–activity relationship (SAR) for the newly synthesised chalcone/HDACI hybrids **(4a-f)** with meta-aromatic cap and hydroxamic acid as ZBG.

First, in comparison of the antiproliferative activity, para orientation of the chalcone moiety generally achieved more favourable cytotoxic activity, for example the unsubstituted analogues revealed that para-substituted compound **5a** displayed substantially improved activity relative to meta-substituted analogue **4a** against DU-145 (2.29 vs 15.91 μM) and PC-3 cells (3.07 vs 20.19 μM). Within the meta series **(4a–f)**, substitution at the aromatic cap significantly enhanced cytotoxic activity compared with the unsubstituted derivative **4a**. The furyl-containing analogue **4b** was the most potent member of this series, exhibiting IC_50_ values of 1.58 and 1.39 μM against DU-145 and PC-3 cells, respectively. Among the halogenated derivatives, activity followed the trend of [Cl > Br > F] against prostate cancer cells, suggesting that increased hydrophobicity and polarizability favour cellular cytotoxic activity. The superior activity of **4d** relative to **4c** may be attributed to the greater lipophilicity and the stronger predicted hydrophobic interactions associated with chlorine substitution. In contrast, the electron-donating methyl derivative **4f** displayed reduced activity, particularly against PC-3 cells, indicating that electron donation alone does not govern cytotoxic potency **(**Figure 12**)**. Within the para series **(5a–f)**, compound **5d** bearing a para-chloro substituent emerged as the most potent analogue against all tested cancer cell lines, with IC_50_ values of 0.93, 1.05, and 2.14 μM against DU-145, PC-3, and MG-63 cells, respectively. The activity trend generally followed the following sequence: [Cl > Furyl > F ≈ CH_3_ > Br] **(**Figure 11**)**. The remarkable potency of **5d** suggests that a moderately electron-withdrawing and hydrophobic substituent provides an optimal balance of electronic and lipophilic properties for cytotoxic activity, in addition to the proper anchoring of the chloro-substituent inside all the HDAC subtypes hydrophobic pockets, in accordance with the docking outcomes. Comparison of corresponding meta and para-analogues further demonstrated that para orientation was particularly advantageous for the chloro derivative **(5d vs 4d)**, producing approximately 2.5-fold and 1.6-fold improvements against DU-145 and PC-3 cells, respectively. This finding suggests that para orientation may promote a more favourable molecular conformation, facilitating improved target engagement. From an electronic perspective, the results do not support a simple electron-donating versus electron-withdrawing relationship. Instead, activity appears to depend on a combination of electronic effects, hydrophobicity, steric factors, and molecular orientation. The superior performance of the chloro derivatives relative to both fluoro and methyl analogues suggests that moderate electron withdrawal combined with increased lipophilicity and size may be beneficial for biological activity. Regarding HDAC inhibition potential, the inhibition activity was markedly influenced by both the aromatic substituent and chalcone orientation. The para-substituted series generally exhibited superior potency, with the chloro-derivative **5d** emerging as the most active compound. Notably, the chloro-substituted analogues (**4d** and **5d**) demonstrated the strongest HDAC inhibition and antiproliferative activities, likely due to their favourable balance of moderate electron-withdrawing character, lipophilicity, and steric properties, which enhance hydrophobic accommodation within the HDAC active site. The superior activity of **5d** over **4d** further suggests that para orientation provides a more favourable tendency for target binding.

## Conclusion

In summary, the antiproliferative activity profile of newly synthesised twelve hydroxamic acid hybrids two series (**4a-f**) and (**5a-f**) *in vitro* against two prostate cancer cell lines (DU-145 and PC-3) and osteosarcoma cells (MG-63) were presented. Hybrid **5d** was the most potent compound with the highest antiproliferative activity against Du-145 (IC_50_ = 0.93 ± 0.1 µM), PC-3 (IC_50_ = 1.05 ± 0.17 µM) and MG-63 (IC_50_ = 2.14 ± 0.45 µM) cell lines. Hybrid **5d** also displayed good apoptotic activity with causing cell cycle arrest of PC3 cells in S phase, whereas it arrests MG-63 cells in G0/G1 phases. Also, it exhibited a potent anti-migratory effect on the three tested cell lines, indicating the potential use of this compound in the advanced prostate cancer stages with bone metastasis. The docking results revealed higher affinity of the most active compound **5d** against HDACs 2, 4, 6 and 8 biotargets, with a great advantage over the SAHA reference drug. Besides, using an atomistic standard 100 ns dynamic simulation consideration, the stability of the formed complexes between compound **5d** and the selected HDACs active sites was considered under dynamic behaviour. Compound **5d** exposed further oral pharmacokinetic advantages over SAHA reference, of being higher lipophilic, lower water soluble and higher drug size within the permissible limits, that support its preference for oral administration.

## Experimental

### Chemistry

All chemicals and organic solvents utilised to synthesise the desired compounds are of commercial grade and purchased from Alfa Aesar, Sigma Aldrich, Merck and El-gomhoria companies for pharmaceutical and used without further purification. Reaction progress was monitored using pre-coated TLC plates (Kiesslgel 60 F254, Merck), detecting spots was done *via* UV lamp at a wavelength of 254 nm. Melting points were measured on Stuart Electro-Thermal apparatus and were uncorrected. ^1^H NMR and ^13^C NMR spectra for all new compounds were recorded in CDCl3 or DMSO-d6 on a Bruker Bio Spin AG spectrometer at 400 MHz and 100 MHz, respectively. For ^1^H NMR, chemical shifts (δ) were given in parts per million (ppm) with reference to tetramethylsilane (TMS) as an internal standard (δ = 0); coupling constants (J) were given in hertz (Hz) and data are reported as follows: chemical shift, integration, multiplicity (s = singlet, d = doublet, m = multiplet). For ^13^C NMR, TMS (δ = 0), DMSO (δ = 39.51) or CDCl_3_ (δ = 77.2) was used as internal standard and spectra were obtained with complete proton decoupling. Elemental analyses were obtained on a Perkin-Elmer CHN-analyzer model. at the regional centre for mycology and biotechnology, Al-Azhar University, Nasr city, Cairo, Egypt.

#### General procedure for synthesis of 7–(3-acetylphenoxy) heptanoic acids 1a or 7–(4-acetylphenoxy) heptanoic acids 1b

A mixture of 3 or 4-hydroxy acetophenone (5 mmol, 0.6 g), anhydrous potassium carbonate (10 mmol, 1.38 g) and ethyl 7-bromoheptanoate ester (5 mmol, 1.4 ml) was stirred in DMF (20 ml) at 70–80 °C for 12 h. as monitored by TLC (MeOH/CHCl_3_, 0.5:9.5),. After cooling to room temperature and acidification with dilute HCl, the separated solid was filtered and washed with water. The crude product was dried and recrystallized from ethyl acetate to afford the pure products **1a** and **1b.**

##### 7–(3-Acetylphenoxy) heptanoic acid (1a)

White powder; Yield (0.62 g, 90%); m.p. 270–272 °C; ^1^H NMR (400 MHz, DMSO-*d_6_*) δ (ppm): 7.54 (d, 1H, Ar-H, *J* = 8), 7.44 (t, 2H, Ar-H, *J* = 8 Hz), 7.21 (d, 1H, Ar-H, *J* = 8 Hz), 4.02 (t, 2H, O-CH_2_, *J* = 8 Hz), 2.58 (s, 3H, -CH_3_), 2.22 (t, 2H, CH_2_-C = O, *J* = 8 Hz), 1.76–1.69 (m, 2H), 1.57–1.49 (m, 2H), 1.47–1.40 (m, 2H), 1.37–1.32 (m, 2H); ^13^C NMR (100 MHz, DMSO-*d_6_*) δ (ppm): 197.4 (C = O), 177.1 (COOH), 159.0 (Ar-C-O), 136.9, 129.2, 122.2, 120.1, 115.0, 69.6, 36.6, 29.0, 28.7, 27.8, 26.5, 24.81; Anal. Calcd. For C_15_H_20_O_4_ (264.32): C, 68.16; H, 7.63 Found: C, 68.23; H, 7.72.

##### 7–(4-Acetylphenoxy) heptanoic acid (1b)^78^

White powder; Yield (0.66 g, 95%); m.p. 281–283 °C; ^1^H NMR (400 MHz, DMSO-*d_6_*) δ (ppm): 7.92 (d, 2H, Ar-H, *J* = 8 Hz), 7.03 (d, 2H, Ar-H, *J* = 8 Hz), 4.05 (t, 2H, O-CH_2_, *J* = 8 Hz), 2.52 (s, 3H, -CH_3_), 2.22 (t, 2H, CH_2_- C = O, *J* = 8 Hz), 1.76–1.69 (m, 2H), 1.56–1.49 (m, 2H), 1.46–1.38 (m, 2H), 1.37–1.31 (m, 2H); ^13^C NMR (100 MHz, DMSO-*d_6_*) δ (ppm): 196.6 (C = O), 174.9 (COOH), 163.0 (Ar-C-O), 130.9, 130.3, 114.7, 68.3, 34.1, 28.8, 28.7, 26.8, 25.6, 24.9; Anal. Calcd. For C_15_H_20_O_4_ (264.32): C, 68.16; H, 7.63 Found: C, 68.26; H, 7.75.

#### General procedure for synthesis of 7–(3-substituted acryloylphenoxy) heptanoic acid chalcones with 6-carbon aliphatic linker 2a-j

A mixture of the 7–(3-Acetylphenoxy)heptanoic acid **1a** (0.26 g, 1 mmol), appropriate aromatic aldehydes (1 mmol) and sodium hydroxide (0.2 g, 5 mmol) in ethanol (20 ml) was stirred at room temperature for 3 h. After completion of the reaction, as monitored by TLC (MeOH/CHCl_3_, 0.5:9.5), conc HCl was added until the PH (1–2). The produced precipitate was filtered, washed with water and dried. Crystallisation from ethanol afforded the target compounds **2a-j**.

##### (E) −7–(3-cinnamoylphenoxy) heptanoic acid (2a)

Yellow powder; Yield (0.55 g, 80%); m.p0.105–110 °C; ^1^H NMR (400 MHz, DMSO-*d_6_*) δ (ppm): 12.04 (s, 1H, -OH), 7.94 (d, 1H, CH = CH, *J* = 16 Hz), 7.90 (d, 2H, Ar-H, *J* = 8 Hz), 7.78–7.74 (m, 2H, Ar-H), 7.63 (s, 1H, Ar- H), 7.49 (d, 2H, Ar-H, *J* = 8 Hz), 7.46 (d, 2H, Ar-H, *J* = 8 Hz), 7.23 (d, 1H, Ar-H, *J* = 8 Hz), 4.05 (t, 2H, O-CH_2_, *J* = 8 Hz), 2.23 (t, 2H, CH_2_-C = O, *J* = 8 Hz), 1.77–1.72 (m, 2H), 1.57–1.50 (m, 2H), 1.48–1.41 (m, 2H), 1.38–1.33 (m, 2H); ^13^C NMR (100 MHz, DMSO-*d_6_*) δ (ppm): 189.3 (C = O), 175.0 (COOH), 159.5 (Ar-C-O), 144.6, 139.4, 135.1, 131.1, 130.4, 129.4, 129.4, 122.5, 121.4, 120.0, 114.1, 68.1 (O-CH_2_), 34.1, 29.0, 28.8, 25.8, 24.9; Anal. Calcd. For C_22_H_24_O_4_ (352.42): C, 74.98; H, 6.86 Found: C, 75.23; H, 6.95.

##### (E)-7–(3-(3-(furan-2-yl)acryloyl) phenoxy) heptanoic acid (2b)

Brown powder; Yield (0.50 g, 75%); m.p. 96–99 °C; ^1^H NMR (400 MHz, DMSO-*d_6_*) δ (ppm): 12.04 (s, 1H, -OH), 7.93 (s, 1H, Ar-H), 7.66 (d, 1H, Ar-H, *J* = 8 Hz), 7.59 (d, 1H, CH = CH, *J* = 16 Hz), 7.53 (d, 2H, Ar-H, *J* = 8 Hz), 7.47 (t, 1H, Ar-H, *J* = 8 Hz), 7.23 (d, 1H, Ar-H, *J* = 8), 7.14 (d, 1H, Ar-H, *J* = 8 Hz), 6.71 (s, 1H, Ar-H), 4.06 (t, 2H, -OCH_2_, *J* = 8 Hz), 2.23 (t, 2H, CH_2_-C = O, *J* = 8 Hz), 1.78–1.73 (m, 2H), 1.56–1.52 (m, 2H), 1.47–1.41 (m, 2H), 1.37–1.35 (m, 2H); ^13^C-NMR (100 MHz, DMSO-*d_6_*) δ (ppm): 188.9 (C = O), 175.0 (COOH), 159.5 (Ar-C-O), 151.6, 146.7, 139.4, 131.0, 130.5, 121.1, 120.1, 119.3, 117.6, 113.7, 113.6, 68.1 (O-CH_2_), 34.1, 29.0, 28.8, 25.7, 24.9; Anal. Calcd. For C_20_H_22_O_5_ (342.39): C, 70.16; H, 6.48 Found: C, 70.42; H, 6.61.

##### (E)-7–(3-(3–(4-fluorophenyl)acryloyl)phenoxy) heptanoic acid (2c)

Yellow powder; Yield (0.41 g, 57%); m.p. 98–100 °C; ^1^H NMR (400 MHz, DMSO-*d_6_*) δ (ppm): 12.10 (s, 1H, -OH), 8.01 (t, 2H, Ar-H, *J* = 8 Hz), 7.92 (d, 1H, CH = CH, *J* = 16 Hz), 7.76 (d, 2H, Ar-H, *J* = 8 Hz), 7.63 (s, 1H, Ar- H), 7.49 (t, 1H, Ar-H, *J* = 8 Hz), 7.32 (t, 2H, Ar-H, *J* = 8 Hz), 7.24 (d, 1H, Ar-H, *J* = 8 Hz), 4.06 (t, 2H, O-CH_2_, *J* = 8 Hz), 2.22 (t, 2H, CH_2_-C = O, *J* = 8 Hz), 1.77–1.73 (m, 2H), 1.56–1.52 (m, 2H), 1.45–1.42 (m, 2H), 1.39–1.35 (m, 2H); ^13^C NMR (100 MHz, DMSO*-d_6_*) δ (ppm): 189.3 (C = O), 175.1 (COOH), 159.5 (Ar-C-O), 143.3, 139.4, 131.9, 131.8, 130.4, 122.4, 121.4, 120.0, 116.5, 116.3, 114.2, 68.1 (O-CH_2_), 34.2, 29.0, 28.8, 25.8, 25.0; Anal. Calcd. For C_22_H_23_FO_4_ (370.41): C, 71.34; H, 6.26 Found: C, 71.53; H, 6.53.

##### (E)-7–(3-(3–(4-Chlorophenyl)acryloyl)phenoxy) heptanoic acid (2d)

White powder; Yield (0.68 g, 90%); m.p0.130–132 °C; ^1^H NMR (400 MHz, DMSO-*d_6_*) δ (ppm): 12.03 (s, 1H, -OH), 7.97–7.90 (m, 3H, Ar-H), 7.76 (d, 1H, Ar-H, *J* = 8 Hz), 7.73 (d, 1H, CH = CH, *J* = 16 Hz), 7.63 (s, 1H, Ar-H), 7.53 (d, 2H, Ar-H, *J* = 8 Hz), 7.48 (t, 1H, Ar-H, *J* = 8 Hz), 7.24 (d, 1H, Ar-H, *J* = 8 Hz), 4.05 (t, 2H, O-CH_2_, *J* = 8 Hz), 2.23 (t, 2H, CH_2_-C = O, *J* = 8 Hz), 1.78–1.71 (m, 2H), 1.57–1.50 (m, 2H), 1.47–1.41 (m, 2H), 1.38–1.33 (m, 2H); ^13^C NMR (100 MHz, DMSO-*d_6_*) δ (ppm): 189.1 (C = O), 175.0 (COOH), 159.7 (Ar-C-O), 143.1, 139.3, 135.6, 134.1, 131.2, 130.4, 129.4, 123.2, 121.4, 120.0, 114.2, 68.1 (O-CH_2_), 34.1, 29.0, 28.8, 25.8, 24.9; Anal. Calcd. For C_22_H_23_ClO_4_ (386.87): C, 68.30; H, 5.99 Found: C, 68.49; H, 6.12.

##### (E)-7–(3-(3–(4-bromophenyl)acryloyl)phenoxy) heptanoic acid (2e)

White powder; Yield (0.76 g, 90%); m.p0.123–127 °C; ^1^H NMR (400 MHz, DMSO-*d_6_*) δ (ppm): 12.04 (s, 1H, -OH), 7.97 (d, 1H, CH = CH, *J* = 16 Hz), 7.87 (d, 2H, Ar-H, *J* = 8 Hz), 7.75 (d, 1H, Ar-H, *J* = 8 Hz), 7.70 (s, 1H, Ar-H), 7.64–7.47 (m, 4H, Ar-H), 7.22 (d, 1H, Ar-H, *J* = 8 Hz), 4.03 (t, 2H, O- CH**_2_**, *J* = 8 Hz), 2.22 (t, 2H, CH**_2_**-C = O, *J* = 8 Hz), 1.76–1.71 (m, 2H), 1.57–1.51 (m, 2H), 1.47–1.42 (m, 2H), 1.37–1.33 (m, 2H); ^13^C NMR (100 MHz, DMSO-*d_6_*) δ (ppm): 189.1 (C = O), 175.0 (COOH), 159.5 (Ar-C-O), 143.1, 139.3, 134.4, 132.3, 131.3, 130.4, 124.5, 123.2, 121.4, 120.0, 114.2, 68.1 (O-CH_2_), 34.1, 29.0, 28.8, 25.8, 24.9; Anal. Calcd. For C_22_H_23_BrO_4_ (431.32): C, 61.26; H, 5.37 Found: C, 61.50; H, 5.63.

##### (E)-7–(3-(3-(p-tolyl)acryloyl)phenoxy)heptanoic acid (2f)

Yellow powder; Yield (0.54 g, 75%); m.p0.98–100 °C; ^1^H NMR (400 MHz, DMSO-*d_6_*) δ (ppm): 12.06 (s, 1H, OH), 7.89 (d, 1H, CH = CH, *J* = 16 Hz), 7.81 (d, 2H, Ar-H, *J* = 8 Hz), 7.73 (m, 2H), 7.62 (s, 1H, Ar-H), 7.48 (t, 1H, Ar-H, *J* = 8 Hz), 7.29 (d, 2H, Ar-H, *J* = 8 Hz), 7.23 (d, 1H, Ar-H, *J* = 8 Hz), 4.06 (t, 2H, O-CH_2_, *J* = 8 Hz), 2.36 (s, 3H, -CH_3_), 2.22 (t, 2H, CH_2_-C = O, *J* = 8 Hz), 1.76–1.73 (m, 2H), 1.56–1.52 (m, 2H), 1.45–1.43 (m, 2H), 1.36–1.35 (m, 2H); ^13^C NMR (100 MHz, DMSO-*d_6_*) δ (ppm): 189.3 (C = O), 175.2 (COOH), 159.5 (Ar-C-O), 144.7, 141.2, 139.5, 132.4, 130.4, 130.0, 129.5, 121.4, 121.3, 119.9, 114.1, 67.8 (O-CH_2_), 34.3, 29.0, 28.8, 25.8, 25.0, 21.5 (-CH_3_); Anal. Calcd. For C_23_H_26_O_4_ (366.45): C, 75.38; H, 7.15 Found: C, 75.54; H, 7.15.

#### General procedure for synthesis of 7–(4 substituted acryloylphenoxy) heptanoic acid chalcones with 6-carbon aliphatic linker 3a-j

A mixture of the 7–(4-acetylphenoxy) heptanoic acid **1b** (0.26 g, 1 mmol), appropriate aromatic aldehydes (1 mmol) and sodium hydroxide (0.2 g, 5 mmol) in ethanol (20 ml) stirred at room temperature for 3 h. After completion of the reaction, as monitored by TLC (MeOH/CHCl_3_, 0.5:9.5), conc HCl was added until the PH (1–2). The produced precipitate was filtered, washed with water and dried. Crystallisation from ethanol afforded the target compounds **3a-j**.

##### (E)-7–(4-cinnamoylphenoxy) heptanoic acid (3a)

Yellow powder; Yield (0.44 g, 65%); m.p0.110–112 °C; ^1^H NMR (400 MHz, DMSO-*d_6_*) δ (ppm): 12.08 (s, 1H, -OH), 8.17 (d, 2H, Ar-H, *J* = 8 Hz), 7.97 (d, 1H, CH = CH, *J* = 16 Hz), 7.89 (d, 2H, Ar-H, *J* = 8 Hz), 7.72 (d, 1H, CH = CH, *J* = 16 Hz), 7.47 (t, 3H, Ar-H, *J* = 8), 7.09 (d, 2H, Ar-H, *J* = 8), 4.09 (t, 2H, O-CH_2_, *J* = 8 Hz), 2.23 (t, 2H, CH_2_-C = O, *J* = 8 Hz), 1.78–1.73 (m, 2H), 1.57–1.50 (m, 2H), 1.47–1.40 (m, 2H), 1.38–1.33 (m, 2H); ^13^C NMR (100 MHz, DMSO-*d_6_*) δ (ppm): 187.8 (C = O), 175.0 (COOH), 163.3 (Ar- C-O), 143.6, 135.3, 131.4, 130.9, 130.7, 129.4, 129.3, 122.5, 114.9, 68.3 (O-CH_2_), 34.1, 28.9, 28.8, 25.7, 24.9; Anal. Calcd. For C_22_H_24_O_4_ (352.42): C, 74.98; H, 6.86, Found: C, 75.19; H, 6.70.

##### (E)-7–(4-(3-(furan-2-yl)acryloyl)phenoxy)heptanoic acid (3b)

Brown powder; Yield (0.51 g, 77%); m.p0.120–122 °C; ^1^H NMR (400 MHz, DMSO-*d_6_*) δ (ppm): 12.03 (s, 1H, -OH), 8.07 (d, 2H, Ar-H, *J* = 8 Hz), 7.91 (s, 1H, Ar-H), 7.56–7.49 (m, 2H, Ar-H), 7.08–6.69 (m, 4H, Ar-H), 4.08 (t, 2H, -OCH_2_, *J* = 8 Hz), 2.23 (t, 2H, -CH_2_-C = O, *J* = 8 Hz), 1.78–1.71 (m, 2H), 1.57–1.52 (m, 2H), 1.47–1.40 (m, 2H), 1.38–1.33 (m, 2H); ^13^C NMR (100 MHz, DMSO-*d_6_*) δ (ppm): 187.2 (C = O), 175.0 (COOH), 163.1 (Ar-C-O), 151.3, 146.4, 131.2, 130.7, 130.2, 119.2, 117.0, 115.0, 113.5, 68.2 (O-CH_2_), 34.1, 28.9, 28.7, 25.6, 24.9.; Anal.Calcd. For C_20_H_22_O_5_ (342.39): C, 70.16; H, 6.48, Found: C, 70.38; H, 6.65.

##### (E)-7–(4-(3–(4-fluorophenyl)acryloyl)phenoxy) heptanoic acid (3c)

Yellow powder; Yield (0.51 g, 70%); m.p0.118–120 °C; ^1^H NMR (400 MHz, DMSO-*d_6_*) δ (ppm): 12.03 (s, 1H, -OH), 8.17–7.98 (m, 4H, Ar-H), 7.93 (d, 1H, CH = CH, *J* = 16 Hz), 7.72 (d, 1H, CH = CH, *J* = 16 Hz), 7.31 (t, 2H, Ar-H, *J* = 8 Hz), 7.08 (d, 2H, Ar-H, *J* = 8 Hz), 4.08 (t, 2H, O-CH_2_, *J* = 8 Hz), 2.23 (t, 2H, CH_2_-C = O, *J* = 8 Hz), 1.76–1.73 (m, 2H), 1.59–1.50 (m, 2H), 1.47–1.40 (m, 2H), 1.36–1.34 (m, 2H); ^13^C NMR (100 MHz, DMSO-*d_6_*) δ (ppm): 187.7 (C = O), 175.0 (COOH), 163.2 (Ar-C-O), 142.4, 132.0, 131.6, 131.4, 130.7, 122.3, 116.5, 116.3, 114.9, 68.4 (O-CH_2_), 34.1, 28.9, 28.8, 25.7, 24.9; Anal. Calcd. For C_22_H_23_FO_4_ (370.41): C, 71.34; H, 6.26, Found: C, 71.57; H, 6.52.

##### (E)-7–(4-(3–(4-Chlorophenyl)acryloyl)phenoxy) 3heptanoic acid (3d)

White powder; Yield (0.6 g, 80%); m.p0.153–155 °C; ^1^H NMR (400 MHz, DMSO-*d_6_*) δ (ppm): 12.32 (s, 1H, -OH), 8.16 (d, 2H, Ar-H, *J* = 8 Hz), 7.98 (d, 1H, CH = CH, *J* = 16 Hz), 7.93 (d, 2H, Ar-H, *J* = 8 Hz), 7.70 (d, 1H, CH = CH, *J* = 16 Hz), 7.52 (d, 2H, Ar-H, *J* = 8 Hz), 7.07 (d, 2H, Ar-H, *J* = 8 Hz), 4.06 (t, 2H, O-CH_2_, *J* = 8 Hz), 2.18 (t, 2H, CH_2_-C = O, *J* = 8 Hz), 1.76–1.70 (m, 2H), 1.56–1.49 (m, 2H), 1.42–1.40 (m, 2H), 1.36–1.33 (m, 2H); ^13^C-NMR (100 MHz, DMSO-*d_6_*) δ (ppm): 187.8 (C = O), 175.3 (COOH), 163.2 (Ar-C-O), 142.1, 135.3, 134.3, 131.5, 131.0, 130.6, 129.4, 123.2, 114.9, 68.3 (O-CH_2_), 34.4, 28.9, 28.8, 25.7, 25.1; Anal. Calcd.For C_22_H_23_ClO_4_ (386.87): C, 68.30; H, 5.99, Found: C, 68.57; H, 5.76.

##### (E)-7–(4-(3–(4-bromophenyl)acryloyl)phenoxy) heptanoic acid (3e)

White powder; Yield (0.72 g, 85%); m.p0.155–157 °C; ^1^H NMR (400 MHz, DMSO-*d_6_*) δ (ppm): 12.13 (s, 1H, -OH), 8.17 (d, 2H, Ar-H, *J* = 8 Hz), 8.00 (d, 1H, CH = CH, *J* = 16 Hz), 7.86 (d, 2H, Ar-H, *J* = 8 Hz), 7.67 (t, 3H, Ar-H, *J* = 8 Hz), 7.08 (d, 2H, Ar-H, *J* = 8 Hz), 4.08 (t, 2H, O-CH_2_, *J* = 8 Hz), 2.23 (t, 2H, CH_2_-C = O, *J* = 8 Hz), 1.76–1.71 (m, 2H), 1.57–1.50 (m, 2H), 1.47–1.41 (m, 2H), 1.38–1.34 (m, 2H); ^13^C NMR (100 MHz, DMSO-*d_6_*) δ (ppm): 187.8 (C = O), 174.9 (COOH), 163.3 (Ar-C-O), 142.2, 134.5, 132.3, 131.5, 131.2, 130.6, 124.2, 123.3, 114.9, 68.3 (O-CH_2_), 34.1, 28.9, 28.8, 25.7, 24.9; Anal. Calcd. For C_22_H_23_BrO_4_ (431.32): C, 61.26; H, 5.37, Found: C, 61.49; H, 5.51.

##### (E)-7–(4-(3-(p-tolyl)acryloyl)phenoxy)heptanoic acid (3f)

Yellow powder; Yield (0.47 g, 66%); m.p0.141–143 °C; ^1^H NMR (400 MHz, DMSO-*d_6_*) δ (ppm): 12.03 (s, 1H, -OH), 8.16 (d, 2H, Ar-H, *J* = 8 Hz), 7.91 (d, 1H, CH = CH, *J* = 16 Hz), 7.79 (d, 2H, Ar-H, *J* = 8 Hz), 7.69 (d, 1H, CH = CH, *J* = 16 Hz), 7.28 (d, 2H, Ar-H, *J* = 8 Hz), 7.08 (d, 2H, Ar-H, *J* = 8 Hz), 4.08 (t, 2H, O-CH_2_, *J* = 8 Hz), 2.36 (s, 3H, -CH_3_), 2.23 (t, 2H, -CH2-C = O, *J* = 8 Hz), 1.76–1.73 (m, 2H), 1.57–1.50 (m, 2H), 1.45–1.40 (m, 2H), 1.36–1.34 (m, 2H); ^13^C NMR (100 MHz, DMSO-*d_6_*) δ (ppm): 187.8 (C = O), 175.0 (COOH), 163.1 (Ar-C-O), 143.7, 141.0, 132.5, 131.4, 130.8, 130.0, 129.28, 121.3, 114.9, 68.3 (O-CH_2_), 34.0, 28.8, 25.7, 24.9, 21.6, 14.6 (CH_3_); Anal. Calcd. For C_23_H_26_O_4_ (366.45): C, 75.38; H, 7.15, Found: C, 75.54; H, 7.15.

#### General procedure for synthesis of 7–(3-substitutedacryloylphenoxy)-N-hydroxyheptanamide derivatives 4a-f

A mixture of chalcone derivatives (**2a-f**) (0.3 g, 1 mmol) and CDI (0.1 g, 1.5 mmol) in acetonitrile (5 ml) was stirred at room temperature for 1–2 h. Hydroxyl amine chloride was added to the reaction mixture and stirring was continuous for a further 3 h. After completion of the reaction, as monitored by TLC (MeOH/CHCl_3_, 0.5:9.5), water was added. The produced precipitate was filtered, washed with water and dried. Crystallisation from ethyl acetate afforded the target compounds **4a-f**.

##### (E)-7–(3-cinnamoylphenoxy)-N-hydroxyheptanamide (4a)

White powder; Yield (0.36 g, 69%); m.p. 120–122 °C; ^1^H NMR (400 MHz, DMSO-*d_6_*) δ (ppm): 10.37 (s, 1H, -NH), 8.69 (s, 1H, -OH), 7.93 (d, 2H, Ar-H, *J* = 8 Hz), 7.77–7.63 (m, 4H, Ar-H), 7.48–7.25 (m, 5H, Ar-H), 4.07 (t, 2H, -OCH_2_, *J* = 8 Hz), 2.22 (t, 2H, -H_2_C-C = O, *J* = 8 Hz), 1.99–1.90 (m, 2H), 1.85–1.74 (m, 2H), 1.60–1.54 (m, 2H), 1.45–1.35 (m, 2H); ^13^C NMR (100 MHz, DMSO-*d_6_*) δ (ppm): 187.9 (C = O), 169.7 (C = O-NH), 163.2 (Ar-C-O), 143.6, 135.3, 131.4, 130.9, 130.8, 129.4, 129.2, 127.8, 127.0, 122.6, 114.9, 68.3 (O-CH), 34.2, 32.7, 28.8, 25.6, 25.0; Anal. Calcd. For C_22_H_25_NO_4_ (367.44): C, 71.91; H, 6.86; N, 3.81. Found: C, 72.07; H, 6.98; N, 4.05.

##### (E)-7–(3-(3-(furan-2-yl)acryloyl)phenoxy)-N-hydroxy heptanamide (4b)

Brown powder; Yield (0.3 g, 57%); m.p0.110–112 °C; ^1^H NMR (400 MHz, DMSO-*d_6_*) δ (ppm): 10.41 (s, 1H, -NH), 9.12 (s, 1H, -OH), 7.94 (s, 1H, Ar-H), 7.69 (d, 1H, CH = CH, *J* = 16), 7.63 (d, 1H, CH = CH, *J* = 16), 7.56 (d, 1H, Ar-H, *J* = 8), 7.51 (d, 1H, Ar-H, *J* = 8), 7.47–7.23 (m, 2H, Ar-H), 7.15 (d, 1H, Ar-H, *J* = 8), 6.71 (s, 1H, Ar-H), 4.06 (t, 2H, -OCH_2_, *J* = 8), 2.23 (t, 2H, -CH_2_-C = O, *J* = 8), 1.76–1.31 (m, 8H); ^13^C-NMR (100 MHz, DMSO-*d_6_*) δ (ppm): 187.3 (C = O), 169.6 (C = O-NH), 163.1 (Ar-C-O), 151.8, 146.3, 131.1, 130.7, 130.2, 119.3, 116.8, 115.0, 113.5, 68.3 (O-CH_2_), 34.3, 32.7, 28.9, 25.6, 25.0; Anal. Calcd. For C_20_H_23_NO_5_ (357.40): C, 67.21; H, 6.49; N, 3.92. Found: C, 67.51; H, 6.69; N, 4.21.

##### (E)-7–(3-(3–(4-fluorophenyl)acryloyl)phenoxy)-N-hydroxy heptanamide (4c)

White powder; Yield (0.25 g, 48%); m.p. 110–112 °C; ^1^H NMR (400 MHz, DMSO-*d_6_*) δ (ppm): 10.41 (s, 1H, -NH), 8.19 (s, 1H, -OH), 8.01 (t, 1H, Ar-H, *J* = 8 Hz), 7.92 (d, 1H, CH = CH, *J* = 16 Hz), 7.77 (d, 1H, CH = CH, *J* = 16 Hz), 7.63 (s, 1H, Ar-H), 7.49 (t, 2H, Ar-H, *J* = 8 Hz), 7.42 (s, 1H, Ar-H), 7.32–7.25 (m, 3H, Ar-H), 4.05 (t, 2H, -OCH_2_, *J* = 8 Hz), 2.20 (t, 2H, -CH_2_- C = O, *J* = 8 Hz), 2.02–1.38 (m, 8H); ^13^C NMR (100 MHz, DMSO-*d_6_*) δ (ppm): 187.8 (C = O), 169.8 (C = O-NH), 159.3 (Ar-C-O), 142.3, 134.4, 132.0, 131.4, 130.7, 129.8, 127.3, 122.5, 119.6, 116.4, 114.9, 68.3 (O-CH_2_), 34.1, 32.6, 28.7, 25.5, 19.0; Anal. Calcd. For C_22_H_24_FNO_4_ (385.43): C, 68.56; H, 6.28; N, 3.63. Found: C, 68.80; H, 6.40; N, 3.79.

##### (E)-7–(3-(3–(4-Chlorophenyl)acryloyl)phenoxy)-N-hydroxy heptanamide (4d)

White powder; Yield (0.5 g, 96%); m.p0.125–127 °C; ^1^H NMR (400 MHz, DMSO-*d_6_*) δ (ppm): 10.38 (s, 1H, -NH), 8.81 (s, 1H, -OH), 7.98 (t, 2H, Ar-H, *J* = 8 Hz), 7.74 (d, 1H, CH = CH, *J* = 16 Hz), 7.63 (s, 1H, Ar-H), 7.54 (d, 2H, Ar-H, *J* = 8 Hz), 7.48 (d, 2H, Ar-H, *J* = 8 Hz), 7.25 (d, 2H, Ar-H, *J* = 8 Hz), 4.06 (t, 2H, -OCH_2_, *J* = 8 Hz), 1.98 (t, 2H, -CH_2_-C = O, *J* = 8 Hz), 1.78–1.71 (m, 2H), 1.56–1.31 (m, 6H); ^13^C NMR (100 MHz, DMSO-*d_6_*) δ (ppm): 189.4 (C = O), 169.3 (C = O-NH), 159.5 (Ar-C-O), 143.0, 139.4, 135.6, 134.1, 131.1, 130.4, 129.4, 123.5, 121.4, 120.0, 114.4, 68.3 (O-CH_2_), 35.9, 32.7, 29.1, 28.8, 25.8; Anal. Calcd. For C_22_H_24_ClNO_4_ (401.88): C, 65.75; H, 6.02; N, 3.49. Found: C, 65.92; H, 6.23; N, 3.75.

##### (E)-7–(3-(3–(4-bromophenyl) acryloyl) phenoxy) -N-hydroxy heptanamide (4e)

White powder; Yield (0.5 g, 96%); m.p0.118–120 °C; ^1^H NMR (400 MHz, DMSO-*d_6_*) δ (ppm): 10.36 (s, 1H, -NH), 8.69 (s, 1H, -OH), 7.99 (d, 1H, CH = CH, *J* = 16 Hz), 7.89 (d, 1H, Ar-H, *J* = 8 Hz), 7.76 (d, 1H, CH = CH, *J* = 16 Hz), 7.69 (t, 1H, Ar-H, *J* = 8 Hz), 7.63 (s, 1H, Ar-H), 7.49 (t, 3H, Ar-H, *J* = 8 Hz), 7.25 (d, 2H, Ar-H, *J* = 8 Hz), 4.06 (t, 2H, -OCH_2_, *J* = 8 Hz), 1.96 (t, 2H, -CH_2_- C = O, *J* = 8 Hz), 1.76–1.71 (m, 2H), 1.56–1.30 (m, 6H); ^13^C NMR (100 MHz, DMSO-*d_6_*) δ (ppm): 189.5 (C = O), 169.3 (C = O-NH), 159.6 (Ar-C-O), 142.1, 139.4, 134.5, 132.3, 131.3, 130.4, 124.4, 123.5, 121.4, 120.1, 114.3, 68.3 (O-CH_2_), 32.7, 29.0, 28.8, 25.7, 25.5; Anal. Calcd. For C_22_H_24_BrNO_4_ (446.33): C, 59.20; H, 5.42; N, 3.14. Found: C, 59.41; H, 5.68; N, 3.39.

##### (E)-N-hydroxy-7–(3-(3-(p-tolyl)acryloyl)phenoxy) heptanamide (4f)

White powder; Yield (0.43 g, 82.6%); m.p. 105–107 °C; ^1^H NMR (400 MHz, DMSO-*d_6_*) δ (ppm): 10.47 (s, 1H, -NH), 8.10 (s, 1H, -OH), 7.96 (s, 1H, Ar-H), 7.88 (d, 1H, CH = CH, *J* = 16 Hz), 7.79 (d, 1H, Ar-H, *J* = 8 Hz), 7.73 (d, 1H, CH = CH, *J* = 16 Hz), 7.61 (s, 1H, Ar-H), 7.46 (d, 1H, Ar-H, *J* = 8 Hz), 7.33–7.23 (m, 4H, Ar-H), 4.03 (t, 2H, -OCH2, *J* = 8 Hz), 2.36 (s, 3H, -CH_3_), 2.14 (t, 2H, -CH_2_-C = O, *J* = 8 Hz), 1.73–1.33 (m, 8H); ^13^C NMR (100 MHz, DMSO-*d_6_*) δ (ppm): 189.2 (C = O), 169.5 (C = O-NH), 159.5 (Ar-C-O), 144.6, 141.2, 139.6, 132.4, 130.4, 130.0, 129.5, 121.5, 121.3, 119.9, 114.2, 68.2 (O-CH_2_), 32.7, 29.0, 28.8, 25.8, 25.6, 21.6 (CH_3_); Anal. Calcd. For C_23_H_27_NO4 (381.46): C, 72.42; H, 7.13; N, 3.67, Found: C, 72.68; H, 7.40; N, 3.90.

#### General procedure for synthesis of 7–(4-substituted acryloylphenoxy)-N-hydroxyheptanamide derivatives 5a-f

A mixture of chalcone derivatives (**3a-f**) (0.3 g, 1 mmol) and CDI (0.1 g, 1.5 mmol) in acetonitrile (5 ml) were stirred at room temperature for 1–2 h. Hydroxyl amine chloride was added to the reaction mixture and stirring continuous for a further 3 h. After completion of the reaction, as monitored by TLC (MeOH/CHCl_3_, 0.5:9.5), water was added. The produced precipitate was filtered, washed with water and dried. Crystallisation from ethyl acetate afforded the target compounds **5a-f**.

##### (E)-7–(4-cinnamoylphenoxy)-N-hydroxyheptanamide (5a)

Yellow powder; Yield (0.4 g, 80%); m.p. 120–123 °C; ^1^H NMR (400 MHz, DMSO-*d_6_*) δ (ppm): 10.43 (s, 1H, -NH), 8.77 (s, 1H, -OH), 8.16 (d, 2H, Ar-H, *J* = 8 Hz), 7.97–7.87 (m, 3H), 7.73 (d, 1H, CH = CH, *J* = 16 Hz), 7.44–7.05 (m, 5H), 4.02 (t, 2H, -OCH_2_, *J* = 8 Hz), 2.21 (t, 2H, -CH_2_-C = O, *J* = 8 Hz), 1.70–1.31 (m, 8H); ^13^C NMR (100 MHz, DMSO-*d_6_*) δ (ppm): 187.9 (C = O), 169.5 (C = O-NH), 163.2 (Ar-C-O), 143.5, 135.3, 131.4, 130.8, 129.4, 129.2, 122.6, 115.7, 114.9, 68.6 (O-CH_2_), 34.1, 32.7, 28.9, 25.6, 24.9; Anal. Calcd. For C_22_H_25_NO_4_ (367.44): C, 71.91; H, 6.86; N, 3.81. Found: C, 72.12; H, 6.94; N, 4.02.

##### (E)-7–(4-(3-(furan-2-yl)acryloyl)phenoxy)-N-hydroxy heptanamide (5b)

Brown powder; Yield (0.3 g, 57%); m.p0.108–110 °C; ^1^H NMR (400 MHz, DMSO-*d_6_*) δ (ppm): 10.31 (s, 1H, -NH), 8.62 (s, 1H, -OH), 8.05 (d, 2H, Ar-H, *J* = 8 Hz), 7.87 (d, 1H, CH = CH, *J* = 16 Hz), 7.54–7.06 (m, 5H, Ar-H), 6.68 (s, 1H, Ar-H), 4.07 (t, 2H, -OCH_2_, *J* = 8 Hz), 2.22 (t, 2H, -CH_2_-C = O, *J* = 8 Hz), 1.74–1.34 (m, 8H); ^13^C NMR (100 MHz, DMSO-*d_6_*) δ (ppm): 187.3 (C = O), 169.6 (C = O-NH), 163.1 (Ar-C-O), 151.7, 146.4, 131.1, 130.7, 130.2, 119.3, 116.8, 115.0, 113.5, 68.4 (O-CH_2_), 32.7, 28.9, 28.8, 25.6, 25.5; Anal. Calcd. For C_20_H_23_NO_5_ (357.40): C, 67.21; H, 6.49; N, 3.92. Found: C, 67.51; H, 6.69; N, 4.15.

##### (E)-7–(4-(3–(4-fluorophenyl)acryloyl)phenoxy)-N-hydroxy heptanamide (5c)

White powder; Yield (0.3 g, 60%); m.p0.105–107 °C; ^1^H NMR (400 MHz, DMSO-*d_6_*) δ (ppm): 10.30 (s, 1H, -NH), 8.61 (s, 1H, -OH), 8.15 (d, 2H, Ar-H, *J* = 8 Hz), 7.96 (d, 1H, CH = CH, *J* = 16 Hz), 7.85 (d, 2H, Ar-H, *J* = 8 Hz), 7.67 (t, 3H, Ar-H, *J* = 8 Hz), 7.08 (d, 2H, Ar-H, *J* = 8 Hz), 4.09 (t, 2H, -OCH_2_, *J* = 8 Hz), 2.22 (t, 2H, -CH_2_-C = O, *J* = 8 Hz), 1.76–1.74 (m, 2H), 1.56–1.53 (m, 2H), 1.44–1.35 (m, 4H); ^13^C NMR (100 MHz, DMSO-*d_6_*) δ (ppm): 189.4 (C = O), 169.7 (C = O-NH), 159.5 (Ar-C-O), 143.1, 134.5, 132.3, 131.3, 130.4, 123.5, 121.4, 120.0, 114.3, 68.2 (O-CH_2_), 35.5, 32.7, 29.0, 25.8, 25.5; Anal. Calcd. For C_22_H_24_FNO_4_ (385.43): C, 68.56; H, 6.28; N, 3.63. Found: C, 68.82; H, 6.37; N, 3.86.

##### (E)-7–(4-(3–(4-Chlorophenyl)acryloyl)phenoxy)-N-hydroxy heptanamide (5d)

Yellow powder; Yield (0.5 g, 96%); m.p0.140–142 °C; ^1^H NMR (400 MHz, DMSO-*d_6_*) δ (ppm): 10.38 (s, 1H, -NH), 8.96 (s, 1H, -OH), 8.18 (d, 2H, Ar-H, *J* = 8), 7.99 (d, 1H, CH = CH, *J* = 16), 7.94 (d, 2H, Ar-H, *J* = 8 Hz), 7.72–7.52 (m, 3H, Ar-H), 7.09 (d, 2H, Ar-H, *J* = 8 Hz), 4.09 (t, 2H, -OCH_2_, *J* = 8 Hz), 1.98 (t, 2H, -CH_2_-C = O, *J* = 8 Hz), 1.76–1.73 (m, 2H), 1.55–1.52 (m, 2H), 1.43–1.32 (m, 4H); ^13^C NMR (100 MHz, DMSO-*d_6_*) δ (ppm): 187.7 (C = O), 169.6 (C = O-NH), 163.3 (Ar-C-O), 142.1, 135.4, 134.3, 131.5, 131.0, 130.7, 129.4, 123.3, 114.9, 68.3 (O-CH_2_), 32.7, 28.9, 28.8, 25.7, 25.5; Anal. Calcd. For C_22_H_24_ClNO4 (401.88): C, 65.75; H, 6.02; N, 3.49. Found: C, 65.84; H, 6.19; N, 3.71; HPLC analysis: Mobile phase (dipotassium hydrogen phosphate buffer [PH 3.2]: ACN, 30:70 respectively) FR 1 ml/min, retention time = 3.01 min; peak area, 95.1% at λ_max_ 254.

##### (E)-7–(4-(3–(4-bromophenyl)acryloyl)phenoxy)-N-hydroxy heptanamide (5e)

White powder; Yield (0.5 g, 98%); m.p. 143–145 °C; ^1^H NMR (400 MHz, DMSO-*d_6_*) δ (ppm): 10.31 (s, 1H, -NH), 8.58 (s, 1H, -OH), 8.15 (d, 2H, Ar-H, *J* = 8 Hz), 7.96 (d, 1H, CH = CH, *J* = 16 Hz), 7.84 (d, 2H, Ar-H, *J* = 8 Hz), 7.67 (t, 3H, Ar-H, *J* = 8 Hz), 7.08 (d, 2H, Ar-H, *J* = 8 Hz), 4.09 (t, 2H, -OCH_2_, *J* = 8 Hz), 2.22 (t, 2H, -CH_2_-C = O, *J* = 8 Hz), 1.75–1.34 (m, 8H); ^13^C NMR (100 MHz, DMSO-*d_6_*) δ (ppm): 187.8 (C = O), 169.6 (C = O-NH), 163.3 (Ar- C-O), 142.1, 134.7, 132.3, 131.4, 131.1, 130.7, 124.2, 123.4, 114.9, 68.4 (O-CH_2_), 34.1, 32.6, 28.9, 25.6, 24.9; Anal. Calcd. For C_22_H_24_BrNO_4_ (446.33): C, 59.20; H, 5.42; N, 3.14. Found: C, 59.47; H, 5.59; N, 3.41.

##### (E)-N-hydroxy-7–(4-(3-(p-tolyl)acryloyl)phenoxy) heptanamide (5f)

White powder; Yield (0.43 g, 82.6%); m.p. 122–125 °C; ^1^H NMR (400 MHz, DMSO-d6) δ (ppm): 10.38 (s, 1H, -NH), 9.04 (s, 1H, -OH), 8.14 (d, 2H, Ar-H, *J* = 8 Hz), 7.87 (d, 1H, CH = CH, *J* = 16 Hz), 7.76 (d, 2H, Ar-H, *J* = 8 Hz), 7.67 (d, 1H, CH = CH, *J* = 16 Hz), 7.27 (d, 2H, Ar-H, *J* = 8 Hz), 7.07 (d, 2H, Ar- H, *J* = 8 Hz), 4.08 (t, 2H, -OCH_2_, *J* = 8 Hz), 2.36 (s, 3H, -CH_3_), 1.97 (t, 2H, -CH_2_-C = O, *J* = 8 Hz), 1.76–1.70 (m, 2H), 1.54–1.51 (m, 2H), 1.44–1.42 (m, 2H), 1.33–1.30 (m, 2H); ^13^C NMR (100 MHz, DMSO-*d_6_*) δ (ppm): 187.8 (C = O), 169.8 (C = O-NH), 162.7 (Ar-C-O), 143.6, 140.9, 132.6, 131.3, 130.8, 130.0, 129.3, 121.4, 114.9, 68.3 (O-CH_2_), 34.4, 32.7, 28.9, 25.7, 25.1, 21.5 (-CH_3_); Anal. Calcd.For C_23_H_27_NO_4_ (381.46): C, 72.42; H, 7.13; N, 3.67, Found: C, 72.25; H, 7.37; N, 3.86.

### Biological studies

#### Materials and methods

##### Materials

Dulbecco’s Modified Eagle Medium (DMEM), penicillin-streptomycin (100x), phosphate-buffered saline (PBS) (100x), and Trypsin/EDTA were purchased from Lonza Group Ltd., Basel, Switzerland. Foetal bovine serum (FBS) (biological source is the blood of bovine fetuses), sulforhodamine-B (SRB), Tris aminomethane base (TRIS), RNAse A, propidium iodide (PI), and acridine orange were purchased from Sigma-Aldrich, Louis, MO, USA. Trichloroacetic acid (TCA) was purchased from (Merck, Germany). The Annexin V-FITC apoptosis detection kit was purchased from Abcam Inc., Cambridge, UK.

##### Methods

###### Cell culture

Human prostate cancer cell lines (DU-145 and PC-3) and Osteosarcoma cell line (MG-63) and human skin fibroblast cells (HSF) as a normal cell model were obtained from Nawah Scientific Inc., (Mokatam, Cairo, Egypt). Cells were cultured in complete DMEM media supplemented with 10% FBS (heat-inactivated foetal bovine serum) and 100 units/mL PS (penicillin/streptomycin) at 37 °C in a humidified 5% (v/v) CO_2_ atmosphere.

###### Cytotoxicity assay

To assess anticancer activity and safety of newly synthesised compounds, cell viability was determined using the SRB (Sulforhodamine B) assay. DU-145, PC-3, MG-63, and HSF cells were seeded (4x10^3^ cells/well) in 96-well plates for 24 h and treated with compounds at 5 log concentrations (0.01, 0.1, 1, 10, and 100 µM) for 72 h. Cells were fixed with 150 µL of 10% TCA for one hour at 4 °C. After rinsing the cells five times with distilled water, the cells were stained with 70 µL SRB solution and incubated in the dark for 10 min at room temperature. Cells were washed with 1% acetic acid and then air-dried overnight. Tris Base (150 µl) was added to dissolve the dye, and the absorbance was measured at 540 nm. Half maximal inhibitory concentrations (IC_50_) values were calculated for each experiment with the Sigma plot program using the Emax model^19^^,^^23^^,^^24^. The selectivity index (SI), which denotes cytotoxic selectivity (i.e., drug safety), was stated as SI = IC_50_ of normal cells divided by IC_50_ of tumour cells^79^^,^^80^.

###### Enzymology (HDAC inhibitory activity assays)^19^^,^^23^^,^^24^

*In vitro* HDAC inhibitory activity for the most potent chalcone/hydroxamic acid target hybrids (**4b**, **4d**, **5b** & **5d**) was evaluated against HDACs 2,4,6 and 8 using commercially validated fluorogenic assay kits (BPS Bioscience, San Diego, CA, USA): Kinetic HDAC2 Assay Kit (Cat. #53002), Fluorogenic HDAC4 Assay Kit (Cat. #50064), Fluorogenic HDAC6 Assay Kit (Cat. #50076–1), and Fluorogenic HDAC8 Assay Kit (Cat. #50068). All assays employed purified human recombinant enzymes at kit-optimized concentrations: HDAC2 (40 ng/reaction), HDAC4 (60 pg/reaction), HDAC6 (35 ng/reaction), and HDAC8 (20 ng/reaction). Isoform-appropriate fluorogenic substrates were used at the following working concentrations: HDAC2 and HDAC6 utilised Fluorogenic HDAC Substrate 3 (Cat. #50037) at 10 µM and 200 µM, respectively, while HDAC4 and HDAC8 utilised Fluorogenic HDAC Substrate Class 2 A (Cat. #50040) at 20 µM. Reactions were performed in a 96-well black microtiter plate in a total volume of 50 µl (HDAC4/6/8) or 100 µl (HDAC2), with DMSO concentration maintained at ≤1%. Following incubation at 37 °C for 30 min, the lysine developer was added and fluorescence was measured at Ex/Em 350–380/440–460 nm. Test compounds were evaluated at five concentrations (100, 10, 1, 0.1, and 0.01 µM) in duplicate technical replicates. IC_50_ values were calculated by nonlinear regression analysis using a sigmoidal dose-response curve fit and expressed as mean ± SEM. IC_50_ were calculated for each compound using GraphPad Prism software.

###### Cell cycle analysis

DU-145, PC-3, and MG-63 cancer cells were treated with a pre-determined IC50 value of **5d** (the most potent compound) for 48h. Cells were trypsinized, washed twice with ice-cold PBS, suspended in 60% ice-cold ethanol for 1 h at 4 °C, and re-washed in PBS. After that, the cells are resuspended in a staining solution (500 μL of PBS with equal volumes (50 μL) of propidium iodide (PI) and RNase A) and incubated for 20 min at 37 °C in the dark. Finally, flow cytometry analyses were performed using the ACEA Novocyte flow cytometer (ACEA Biosciences Inc., San Diego, USA). Data from 12,000 cells were collected for each sample, and the ACEA Novo Express program (ACEA Biosciences Inc., San Diego, USA) analysed the distribution of cell cycle phases^19^^,^^23^^,^^24^.

###### Apoptosis analysis

After 48 h of **5d** treatment, the cell populations of apoptosis and necrosis in DU-145, PC-3, and MG-63 cells were assessed using the Annexin V-FITC apoptosis detection kit (Abcam Inc., Cambridge Science Park, Cambridge, UK) coupled with flow cytometry. Cells were trypsinized, washed twice with ice-cold PBS, and incubated for 15 min at room temperature in the dark in 0.5 ml of the binding buffer with 5 µL Annexin V-FITC and 5 µL PI solution, according to the manufacturer’s procedure. Following staining, the cells were applied to FACS analysis using an ACEA Novocyte flow cytometer (ACEA Biosciences Inc., San Diego, CA, USA) within 1 h of staining^19^^,^^23^^,^^24^.

###### Cell scratch assay (migration assay)

DU-145, PC-3, and MG-63 cells were seeded at 90% confluency in 12-well plates in triplicate. After 24 h, a scratch was introduced across the centre of each well using a 1 mm pipette tip. The cells were washed twice with PBS to remove cell debris and replaced with fresh media containing the **5d** compound. The wound width is measured by the average distance between the edges of the scratches, which decreases as cell migration is stimulated. Images were captured at 0, 24, 48, 72, 96 and 120 h using an inverted microscope. The wound widths were measured using Image J software version 1.43 m. % migration = (average of scratch width at 0 (h) - average of scratch width at t (h))/average of scratch width at 0 (h) × 100%^81^.

###### Statistical analysis

The data from biological assays including IC_50_ calculations were analysed using the Sigma plot program using the Emax model. The statistical significance of variations in each parameter between the groups was determined using one-way ANOVA, followed by the LSD test. All data are presented as mean ± SD. The significance level was set to *P* < 0.05.

## Docking studies

### Docking methodology

Using the Maestro bundle of Schrodinger software 2024.1, the most stable pose of the most active candidate **5d** was docked against HDAC2 (pdb: 4LXZ)^65^, HDAC4 (pdb: 4CBT)^68^, HDAC6 (pdb: 5EFH)^82^ and HDAC8 (pdb: 3SFH)^73^ biotargets, to approve their true binding. The indicated biotargets were recovered from the RCSB protein data bank (PDB). However, all the mandatory stages for ligand preparation, protein preparation, receptor grid generation and docking process, were illustrated in detail at ***(the supplementary data section)*** as reported^83–86^.

### Molecular dynamic simulations

The stability of the most active compound **5d** along with the reference ligand SAHA co-crystallized with the active sites of HDAC 2, 4, 6, and 8, was assessed through molecular dynamics (MD) simulations. These simulations were performed using the OPLS3e force field within the Desmond software (Desmond 2024–1, Schrödinger LLC)^29^^,^^87^^,^^88^. The MD simulation process consisted of several essential steps, including system setup, charge neutralisation *via* Desmond software, and molecular dynamics optimisation under standard temperature and pressure conditions, are listed in **(**the Supplementary data section**)**, as reported^89–92^.

## Supplementary Material

Supporting_Information__.docx

## Data Availability

The data that support the findings of this study are available from the corresponding author (Mamdouh F. A. Mohamed), upon reasonable request.
