# Supplementary material for: Exploration of novel hydroxamic acid-based candidates integrating chalcone scaffold cap as multitarget HDAC inhibitors: design, anti-prostatic cancer assessment, in silico docking and molecular dynamic simulation
Source: J Enzyme Inhib Med Chem. 2026 Aug 27;41(1):2711875. doi: 10.1080/14756366.2026.2711875 (PMC13523115; doi:10.1080/14756366.2026.2711875)
Supplement: Supplementary file 1 [file IENZ_A_2711875_SM7801.docx]

**Supporting Information**

Exploration of novel hydroxamic acid-based candidates integrating chalcone scaffold cap as multitarget HDAC inhibitors: Design, anti-prostatic cancer assessment, *in silico* docking and molecular dynamic simulation

**Experimental**

**1) Docking Analysis**

Using the Maestro bundle of Schrodinger software 2024.1, the most stable pose of the most active candidate **5d** was docked against HDAC2 (pdb: 4LXZ), HDAC4 (pdb: 4CBT), HDAC6 (pdb: 5EFH) and HDAC8 (pdb: 3SFH) biotargets, were constructed using Chemdraw 18.0 PerkinElmer software and subjected to clean up process for bond alignment then imported to the Maestro software in a 3D representation way. Energy minimization was performed using OPLS3e (Optimized Potentials for Liquid Simulations) force field in Ligprep (Version 2024-1, Schrodinger)[1], that helps to disperse the bond orders. The permitted protonation to the explored hits were executed, then the required output files that denote the best poses of the ligands, were used for vitual consideration. Also, the chosen biotargets preparation was carried out using Protein preparation wizard (Version 2024-1, Schrodinger) [2] as a prime tool for protein preparation. The protein protonation was accomplished, the charges were sanctioned using Epik at pH 7.0 ± 2.0. The desired proteins were subjected to Pre-process, refinement and protein modification by the applicable chain selection, water molecules were eliminated and to finish, the proteins minimization was performed using OPLS3 force field. Likewise, the receptor grids were built by taking in consideration the co-crystal ligands. The ligands centroid was designated to create the grid box around it and Van der Waals radius of receptor atoms was struggled to 10 Å with a partial atomic charge of 0.25. The docking procedure was achieved using rigid docking protocol [3] utilizing Maestro software in Schrodinger. The docking calculations were functioned using Extra Precision (XP) mode, where a scaling factor of 0.8 and a partial atomic charge ˂ 0.15 were concerned to the protein atoms. The glide docking results were retained to validate the best-docked poses from the output and the main interactions of these poses were further investigated using XP visualizer. Lastly, the root mean square deviation (RMSD) was certified to authorize the protein, and the RMSD values presented within 0.46 Å.

**2) Molecular Dynamic Simulation**

The stability of the most active compound **5d** along with the reference ligand SAHA co-crystallized with the active sites of HDAC 2, 4, 6, and 8, was assessed through molecular dynamics (MD) simulations. These simulations were performed using the OPLS3e force field within the Desmond software (Desmond 2024–1, Schrödinger LLC). The ligand–protein complex was resolved in a solvent-saturated orthorhombic periodic box with the least distance of 10 Å between the atoms of protein and the margins of box. The solvent was satisfied using a single point charge water model (OPLS3). The neutralization of the system charge was achieved by contributing Na^+^ and Cl^-^ counter ions and 0.15 M NaCl salt concentration, that was recognized steady with the physiological system using the Desmond *System Builder* panel. Besides, the assembled solvated system was subjected to minimization and relaxation using OPLS4e force field restraints as the default protocol associated with Desmond. The isothermal isobaric collaborative (Normal pressure and temperature/NPT) was considered throughout the simulation, with a temperature of 300k and an atmospheric pressure of 1.0315 bar using the Nose-Hoover chain thermostat method and the Martyna-Tobias-Klein barostat method, respectively. The simulation running was functioned for a period of 100 ns and the gathered MD trajectories were purposed using Desmond’s Simulation Interaction Diagram (SID) to speculate the possible alignments of the ligands. The created MD simulation trajectories were exploited to compute numerous deliberations including the protein Cα atoms Root mean square deviation (RMSD), the RMSD of ligand atoms, protein Root mean square fluctuation (RMSF), and protein ligand contact histogram mapping. The RMSD assumption in the MD simulations gives an estimate to the stability of the protein–ligand complexes and their dynamic performance. The protein structure deviation or the protein-ligand complex deviations from their initial poses were considered by RMSD, that justify the stability of the protein–ligand complex during the simulation time.

**3) Docking Validation:**

| **Target** | **Validation (RMSD)** |
| --- | --- |
| **HDAC2 (pdb: 4LXZ)** |   **RMSD= 0.4987 Å** |
| **HDAC4 (pdb: 4CBT)** |   **RMSD= 0.4062 Å** |
| **HDAC6 (pdb: 5EFH)** |   **RMSD= 1.9746 Å** |
| **HDAC8 (pdb: 3SFH)** |   **RMSD= 1.9461 Å** |

**Spectral data**

7-**(3-Acetylphenoxy) heptanoic acid 1a**

**7-(4-acetylphenoxy) heptanoic acid 1b**

**(*E*) -7-(3-Cinnamoylphenoxy) heptanoic acid 2a:**

**(*E*)-7-(3-(3-(Furan-2-yl)acryloyl) phenoxy) heptanoic acid 2b:**

**(*E*)-7-(3-(3-(4-Fluorophenyl)acryloyl)phenoxy) heptanoic acid 2c:**

**(*E*)-7-(3-(3-(4-Chlorophenyl)acryloyl)phenoxy) heptanoic acid 2d:**

**(*E*)-7-(3-(3-(4-Bromophenyl)acryloyl)phenoxy) heptanoic acid 2e:**

**(*E*)-7-(3-(3-(p-Tolyl)acryloyl)phenoxy)heptanoic acid 2f:**

**(*E*)-7-(4-Cinnamoylphenoxy) heptanoic acid 3a:**

**(*E*)-7-(4-(3-(Furan-2-yl)acryloyl)phenoxy)heptanoic acid 3b:**

**(*E*)-7-(4-(3-(4-Fluorophenyl)acryloyl)phenoxy) heptanoic acid 3c:**

**(*E*)-7-(4-(3-(4-Chlorophenyl)acryloyl)phenoxy) 3heptanoic acid 3d:**

**(*E*)-7-(4-(3-(4-Bromophenyl)acryloyl)phenoxy) heptanoic acid 3e:**

**(*E*)-7-(4-(3-(p-Tolyl)acryloyl)phenoxy)heptanoic acid 3f:**

**(*E*)-7-(3-Cinnamoylphenoxy)-N-hydroxyheptanamide 4a:**

**(*E*)-7-(3-(3-(Furan-2-yl)acryloyl)phenoxy)-N-hydroxy** **heptanamide 4b:**

**(*E*)-7-(3-(3-(4-Fluorophenyl)acryloyl)phenoxy)-N-hydroxy heptanamide 4c:**

**(*E*)-7-(3-(3-(4-Chlorophenyl)acryloyl)phenoxy)-N-hydroxy heptanamide 4d:**

**(*E*)-7-(3-(3-(4-Bromophenyl) acryloyl) phenoxy) -N-hydroxy heptanamide 4e:**

**(*E*)-N-Hydroxy-7-(3-(3-(p-Tolyl)acryloyl)phenoxy) heptanamide 4f:**

**(*E*)-7-(4-Cinnamoylphenoxy)-N-hydroxyheptanamide 5a:**

**(*E*)-7-(4-(3-(Furan-2-yl)acryloyl)phenoxy)-N-hydroxy heptanamide 5b:**

**(*E*)-7-(4-(3-(4-Fluorophenyl)acryloyl)phenoxy)-N-hydroxy heptanamide 5c:**

**(*E*)-7-(4-(3-(4-Chlorophenyl)acryloyl)phenoxy)-N-hydroxy heptanamide 5d:**

**(*E*)-7-(4-(3-(4-Bromophenyl)acryloyl)phenoxy)-N-hydroxy heptanamide 5e:**

**(*E*)-N-Hydroxy-7-(4-(3-(p-Tolyl)acryloyl)phenoxy) heptanamide 5f:**

**HPLC purity chromatograph of compound 5d**

**Statistical figures for cytotoxicity**

**IC_50_ Curves**

**HDAC enzyme inhibition assay**

| **ser** | **Compound** | | **HDACx** | | | | ± |
| --- | --- | --- | --- | --- | --- | --- | --- |
|  | **code** | **MW**  **g/mol** | **IC50**  **uM** | | | |  |
|  |  |  | **HDAC 2** | **HDAC 4** | **HDAC 6** | **HDAC 8** |  |
| 1 | **4d** | **401.88** | 0.949±0.042 | 0.396±0.016 | 1.295±0.05 | 2.476±0.103 |  |
| 2 | **4b** | **355.43** | 3.365±0.148 | 1.782±0.072 | 2.972±0.12 | 3.967±0.165 |  |
| 3 | **5d** | **401.88** | 0.857±0.038 | 0.165±0.007 | 1.171±0.045 | 0.15±0.006 |  |
| 4 | **5b** | **355.43** | 0.572±0.025 | 0.683±0.027 | 0.268±0.01 | 0.881±0.037 |  |
| * | **Saha** | **264.32** | 0.119 | 4.062 | 0.019 | 1.133 |  |

**Detailed results**

| **HDAC2** |  |  |  |  |  |  |  |  |  |  |  |  |
| --- | --- | --- | --- | --- | --- | --- | --- | --- | --- | --- | --- | --- |
| code | IC50 | conc | log | %inh | T2 | T1 | ∆T | RFU2 | RFU1 | ∆RFU | slope | K.Activity |
| 1m |  | 100 | 2 | 89 | 30 | 0 | 30 | 11.43 | 0 | 11.43 | 3.33333 | 13.71601 |
|  |  | 10 | 1 | 74 | 30 | 0 | 30 | 25.99 | 0 | 25.99 | 3.33333 | 31.18803 |
|  |  | 1 | 0 | 53 | 30 | 0 | 30 | 47.03 | 0 | 47.03 | 3.33333 | 56.43606 |
|  |  | 0.1 | -1 | 25 | 30 | 0 | 30 | 75.19 | 0 | 75.19 | 3.33333 | 90.22809 |
|  |  | 0.01 | -2 | 12 | 30 | 0 | 30 | 88.13 | 0 | 88.13 | 3.33333 | 105.7561 |
| EC |  |  |  | 0 | 30 | 0 | 30 | 100 | 0 | 100 | 3.33333 | 120 |
|  |  |  |  |  |  |  |  |  |  |  |  |  |
| code | IC50 | conc | log | %inh | T2 | T1 | ∆T | RFU2 | RFU1 | ∆RFU | slope | K.Activity |
| 8m |  | 100 | 2 | 85 | 30 | 0 | 30 | 14.51 | 0 | 14.51 | 3.33333 | 17.41202 |
|  |  | 10 | 1 | 59 | 30 | 0 | 30 | 41.39 | 0 | 41.39 | 3.33333 | 49.66805 |
|  |  | 1 | 0 | 30 | 30 | 0 | 30 | 69.54 | 0 | 69.54 | 3.33333 | 83.44808 |
|  |  | 0.1 | -1 | 18 | 30 | 0 | 30 | 82.33 | 0 | 82.33 | 3.33333 | 98.7961 |
|  |  | 0.01 | -2 | 4.1 | 30 | 0 | 30 | 95.92 | 0 | 95.92 | 3.33333 | 115.1041 |
| EC |  |  |  | 0 | 30 | 0 | 30 | 100 | 0 | 100 | 3.33333 | 120 |
|  |  |  |  |  |  |  |  |  |  |  |  |  |
| code | IC50 | conc | log | %inh | T2 | T1 | ∆T | RFU2 | RFU1 | ∆RFU | slope | K.Activity |
| 2p |  | 100 | 2 | 90 | 30 | 0 | 30 | 9.53 | 0 | 9.53 | 3.33333 | 11.43601 |
|  |  | 10 | 1 | 69 | 30 | 0 | 30 | 31.06 | 0 | 31.06 | 3.33333 | 37.27204 |
|  |  | 1 | 0 | 48 | 30 | 0 | 30 | 52.19 | 0 | 52.19 | 3.33333 | 62.62806 |
|  |  | 0.1 | -1 | 31 | 30 | 0 | 30 | 68.75 | 0 | 68.75 | 3.33333 | 82.50008 |
|  |  | 0.01 | -2 | 18 | 30 | 0 | 30 | 82.33 | 0 | 82.33 | 3.33333 | 98.7961 |
| EC |  |  |  | 0 | 30 | 0 | 30 | 100 | 0 | 100 | 3.33333 | 120 |
|  |  |  |  |  |  |  |  |  |  |  |  |  |
| code | IC50 | conc | log | %inh | T2 | T1 | ∆T | RFU2 | RFU1 | ∆RFU | slope | K.Activity |
| 8p |  | 100 | 2 | 88 | 30 | 0 | 30 | 12.44 | 0 | 12.44 | 3.33333 | 14.92801 |
|  |  | 10 | 1 | 73 | 30 | 0 | 30 | 26.96 | 0 | 26.96 | 3.33333 | 32.35203 |
|  |  | 1 | 0 | 52 | 30 | 0 | 30 | 47.93 | 0 | 47.93 | 3.33333 | 57.51606 |
|  |  | 0.1 | -1 | 36 | 30 | 0 | 30 | 63.56 | 0 | 63.56 | 3.33333 | 76.27208 |
|  |  | 0.01 | -2 | 21 | 30 | 0 | 30 | 78.62 | 0 | 78.62 | 3.33333 | 94.34409 |
| EC |  |  |  | 0 | 30 | 0 | 30 | 100 | 0 | 100 | 3.33333 | 120 |
|  |  |  |  |  |  |  |  |  |  |  |  |  |
| code | IC50 | conc | log | %inh | T2 | T1 | ∆T | RFU2 | RFU1 | ∆RFU | slope | K.Activity |
| **SAHA** **saha** |  | 10 | 1 | 89 | 30 | 0 | 30 | 11.46 | 0 | 11.46 | 3.333 | 13.753 |
|  |  | 1 | 0 | 70 | 30 | 0 | 30 | 29.53 | 0 | 29.53 | 3.333 | 35.44 |
|  |  | 0.1 | -1 | 47 | 30 | 0 | 30 | 52.79 | 0 | 52.79 | 3.333 | 63.354 |
|  |  | 0.01 | -2 | 28 | 30 | 0 | 30 | 71.61 | 0 | 71.61 | 3.333 | 85.941 |
|  |  |  |  | 0 | 30 | 0 | 30 | 100 | 0 | 100 | 3.333 | 120 |
|  |  |  |  |  |  |  |  |  |  |  |  |  |
|  |  |  |  |  |  |  |  |  |  |  |  |  |
| **HDAC8** |  |  |  |  |  |  |  |  |  |  |  |  |
| code | IC50 | conc | log | %inh | T2 | T1 | ∆T | RFU2 | RFU1 | ∆RFU | slope | K.Activity |
| **1m** |  | 100 | 2 | 92 | 30 | 0 | 30 | 8.03 | 0 | 8.03 | 3.33333 | 9.63601 |
|  |  | 10 | 1 | 78 | 30 | 0 | 30 | 22.46 | 0 | 22.46 | 3.33333 | 26.95203 |
|  |  | 1 | 0 | 63 | 30 | 0 | 30 | 36.71 | 0 | 36.71 | 3.33333 | 44.05204 |
|  |  | 0.1 | -1 | 40 | 30 | 0 | 30 | 59.83 | 0 | 59.83 | 3.33333 | 71.79607 |
|  |  | 0.01 | -2 | 15 | 30 | 0 | 30 | 84.62 | 0 | 84.62 | 3.33333 | 101.5441 |
| EC |  |  |  | 0 | 30 | 0 | 30 | 100 | 0 | 100 | 3.33333 | 120 |
|  |  |  |  |  |  |  |  |  |  |  |  |  |
| code | IC50 | conc | log | %inh | T2 | T1 | ∆T | RFU2 | RFU1 | ∆RFU | slope | K.Activity |
| **8m** |  | 100 | 2 | 88 | 30 | 0 | 30 | 11.53 | 0 | 11.53 | 3.33333 | 13.83601 |
|  |  | 10 | 1 | 72 | 30 | 0 | 30 | 27.92 | 0 | 27.92 | 3.33333 | 33.50403 |
|  |  | 1 | 0 | 38 | 30 | 0 | 30 | 62.31 | 0 | 62.31 | 3.33333 | 74.77207 |
|  |  | 0.1 | -1 | 16 | 30 | 0 | 30 | 84.19 | 0 | 84.19 | 3.33333 | 101.0281 |
|  |  | 0.01 | -2 | 8.9 | 30 | 0 | 30 | 91.06 | 0 | 91.06 | 3.33333 | 109.2721 |
| EC |  |  |  | 0 | 30 | 0 | 30 | 100 | 0 | 100 | 3.33333 | 120 |
|  |  |  |  |  |  |  |  |  |  |  |  |  |
| code | IC50 | conc | log | %inh | T2 | T1 | ∆T | RFU2 | RFU1 | ∆RFU | slope | K.Activity |
| **2p** |  | 100 | 2 | 94 | 30 | 0 | 30 | 6.27 | 0 | 6.27 | 3.33333 | 7.524008 |
|  |  | 10 | 1 | 84 | 30 | 0 | 30 | 15.83 | 0 | 15.83 | 3.33333 | 18.99602 |
|  |  | 1 | 0 | 63 | 30 | 0 | 30 | 37.29 | 0 | 37.29 | 3.33333 | 44.74804 |
|  |  | 0.1 | -1 | 46 | 30 | 0 | 30 | 53.93 | 0 | 53.93 | 3.33333 | 64.71606 |
|  |  | 0.01 | -2 | 29 | 30 | 0 | 30 | 71.11 | 0 | 71.11 | 3.33333 | 85.33209 |
| EC |  |  |  | 0 | 30 | 0 | 30 | 100 | 0 | 100 | 3.33333 | 120 |
|  |  |  |  |  |  |  |  |  |  |  |  |  |
| code | IC50 | conc | log | %inh | T2 | T1 | ∆T | RFU2 | RFU1 | ∆RFU | slope | K.Activity |
| **8p** |  | 100 | 2 | 91 | 30 | 0 | 30 | 8.59 | 0 | 8.59 | 3.33333 | 10.30801 |
|  |  | 10 | 1 | 76 | 30 | 0 | 30 | 24.02 | 0 | 24.02 | 3.33333 | 28.82403 |
|  |  | 1 | 0 | 53 | 30 | 0 | 30 | 47.18 | 0 | 47.18 | 3.33333 | 56.61606 |
|  |  | 0.1 | -1 | 30 | 30 | 0 | 30 | 69.53 | 0 | 69.53 | 3.33333 | 83.43608 |
|  |  | 0.01 | -2 | 16 | 30 | 0 | 30 | 84.36 | 0 | 84.36 | 3.33333 | 101.2321 |
| EC |  |  |  | 0 | 30 | 0 | 30 | 100 | 0 | 100 | 3.33333 | 120 |
|  |  |  |  |  |  |  |  |  |  |  |  |  |
| code | IC50 | conc | log | %inh | T2 | T1 | ∆T | RFU2 | RFU1 | ∆RFU | slope | K.Activity |
| **SAHA** |  | 100 | 2 | 88 | 30 | 0 | 30 | 11.65 | 0 | 11.65 | 3.333 | 13.981 |
|  |  | 10 | 1 | 66 | 30 | 0 | 30 | 33.75 | 0 | 33.75 | 3.333 | 40.504 |
|  |  | 1 | 0 | 49 | 30 | 0 | 30 | 51.04 | 0 | 51.04 | 3.333 | 61.254 |
|  |  | 0.1 | -1 | 30 | 30 | 0 | 30 | 69.51 | 0 | 69.51 | 3.333 | 83.42 |
| EC |  |  |  | 0 | 30 | 0 | 30 | 100 | 0 | 100 | 3.33333 | 120 |
|  |  |  |  |  |  |  |  |  |  |  |  |  |
|  |  |  |  |  |  |  |  |  |  |  |  |  |
| **HDAC4** |  |  |  |  |  |  |  |  |  |  |  |  |
| code | IC50 | conc | log | %inh | T2 | T1 | ∆T | RFU2 | RFU1 | ∆RFU | slope | K.Activity |
| **1m** |  | 100 | 2 | 89 | 30 | 0 | 30 | 11.06 | 0 | 11.06 | 3.33333 | 13.27201 |
|  |  | 10 | 1 | 76 | 30 | 0 | 30 | 23.79 | 0 | 23.79 | 3.33333 | 28.54803 |
|  |  | 1 | 0 | 44 | 30 | 0 | 30 | 55.82 | 0 | 55.82 | 3.33333 | 66.98407 |
|  |  | 0.1 | -1 | 21 | 30 | 0 | 30 | 79.16 | 0 | 79.16 | 3.33333 | 94.99209 |
|  |  | 0.01 | -2 | 7.6 | 30 | 0 | 30 | 92.41 | 0 | 92.41 | 3.33333 | 110.8921 |
| EC |  |  |  | 0 | 30 | 0 | 30 | 100 | 0 | 100 | 3.33333 | 120 |
|  |  |  |  |  |  |  |  |  |  |  |  |  |
| code | IC50 | conc | log | %inh | T2 | T1 | ∆T | RFU2 | RFU1 | ∆RFU | slope | K.Activity |
| **8m** |  | 100 | 2 | 83 | 30 | 0 | 30 | 17.23 | 0 | 17.23 | 3.33333 | 20.67602 |
|  |  | 10 | 1 | 63 | 30 | 0 | 30 | 36.59 | 0 | 36.59 | 3.33333 | 43.90804 |
|  |  | 1 | 0 | 35 | 30 | 0 | 30 | 64.96 | 0 | 64.96 | 3.33333 | 77.95208 |
|  |  | 0.1 | -1 | 16 | 30 | 0 | 30 | 84.03 | 0 | 84.03 | 3.33333 | 100.8361 |
|  |  | 0.01 | -2 | 4.6 | 30 | 0 | 30 | 95.37 | 0 | 95.37 | 3.33333 | 114.4441 |
| EC |  |  |  | 0 | 30 | 0 | 30 | 100 | 0 | 100 | 3.33333 | 120 |
|  |  |  |  |  |  |  |  |  |  |  |  |  |
| code | IC50 | conc | log | %inh | T2 | T1 | ∆T | RFU2 | RFU1 | ∆RFU | slope | K.Activity |
| **2p** |  | 100 | 2 | 86 | 30 | 0 | 30 | 13.52 | 0 | 13.52 | 3.33333 | 16.22402 |
|  |  | 10 | 1 | 70 | 30 | 0 | 30 | 29.91 | 0 | 29.91 | 3.33333 | 35.89204 |
|  |  | 1 | 0 | 43 | 30 | 0 | 30 | 57.18 | 0 | 57.18 | 3.33333 | 68.61607 |
|  |  | 0.1 | -1 | 28 | 30 | 0 | 30 | 72.06 | 0 | 72.06 | 3.33333 | 86.47209 |
|  |  | 0.01 | -2 | 16 | 30 | 0 | 30 | 83.59 | 0 | 83.59 | 3.33333 | 100.3081 |
| EC |  |  |  | 0 | 30 | 0 | 30 | 100 | 0 | 100 | 3.33333 | 120 |
|  |  |  |  |  |  |  |  |  |  |  |  |  |
| code | IC50 | conc | log | %inh | T2 | T1 | ∆T | RFU2 | RFU1 | ∆RFU | slope | K.Activity |
| **8p** |  | 100 | 2 | 92 | 30 | 0 | 30 | 7.53 | 0 | 7.53 | 3.33333 | 9.036009 |
|  |  | 10 | 1 | 78 | 30 | 0 | 30 | 21.84 | 0 | 21.84 | 3.33333 | 26.20803 |
|  |  | 1 | 0 | 65 | 30 | 0 | 30 | 34.63 | 0 | 34.63 | 3.33333 | 41.55604 |
|  |  | 0.1 | -1 | 40 | 30 | 0 | 30 | 59.56 | 0 | 59.56 | 3.33333 | 71.47207 |
|  |  | 0.01 | -2 | 24 | 30 | 0 | 30 | 76.28 | 0 | 76.28 | 3.33333 | 91.53609 |
| EC |  |  |  | 0 | 30 | 0 | 30 | 100 | 0 | 100 | 3.33333 | 120 |
|  |  |  |  |  |  |  |  |  |  |  |  |  |
| code | IC50 | conc | log | %inh | T2 | T1 | ∆T | RFU2 | RFU1 | ∆RFU | slope | K.Activity |
| **SAHA** |  | 100 | 2 | 82 | 30 | 0 | 30 | 18.03 | 0 | 18.03 | 3.333 | 21.638 |
|  |  | 10 | 1 | 60 | 30 | 0 | 30 | 39.91 | 0 | 39.91 | 3.333 | 47.897 |
|  |  | 1 | 0 | 33 | 30 | 0 | 30 | 67.15 | 0 | 67.15 | 3.333 | 80.588 |
|  |  | 0.1 | -1 | 15 | 30 | 0 | 30 | 84.79 | 0 | 84.79 | 3.333 | 101.76 |
| EC |  |  |  | 0 | 30 | 0 | 30 | 100 | 0 | 100 | 3.33333 | 120 |
|  |  |  |  |  |  |  |  |  |  |  |  |  |
|  |  |  |  |  |  |  |  |  |  |  |  |  |
| **HDAC6** |  |  |  |  |  |  |  |  |  |  |  |  |
| code | IC50 | conc | log | %inh | T2 | T1 | ∆T | RFU2 | RFU1 | ∆RFU | slope | K.Activity |
| **1m** |  | 100 | 2 | 87 | 30 | 0 | 30 | 13.42 | 0 | 13.42 | 3.33333 | 16.10402 |
|  |  | 10 | 1 | 62 | 30 | 0 | 30 | 38.06 | 0 | 38.06 | 3.33333 | 45.67205 |
|  |  | 1 | 0 | 37 | 30 | 0 | 30 | 63.29 | 0 | 63.29 | 3.33333 | 75.94808 |
|  |  | 0.1 | -1 | 19 | 30 | 0 | 30 | 81.06 | 0 | 81.06 | 3.33333 | 97.2721 |
|  |  | 0.01 | -2 | 5.4 | 30 | 0 | 30 | 94.61 | 0 | 94.61 | 3.33333 | 113.5321 |
| EC |  |  |  | 0 | 30 | 0 | 30 | 100 | 0 | 100 | 3.33333 | 120 |
|  |  |  |  |  |  |  |  |  |  |  |  |  |
| code | IC50 | conc | log | %inh | T2 | T1 | ∆T | RFU2 | RFU1 | ∆RFU | slope | K.Activity |
| **8m** |  | 100 | 2 | 80 | 30 | 0 | 30 | 19.52 | 0 | 19.52 | 3.33333 | 23.42402 |
|  |  | 10 | 1 | 57 | 30 | 0 | 30 | 42.72 | 0 | 42.72 | 3.33333 | 51.26405 |
|  |  | 1 | 0 | 33 | 30 | 0 | 30 | 67.02 | 0 | 67.02 | 3.33333 | 80.42408 |
|  |  | 0.1 | -1 | 16 | 30 | 0 | 30 | 84.32 | 0 | 84.32 | 3.33333 | 101.1841 |
|  |  | 0.01 | -2 | 7.4 | 30 | 0 | 30 | 92.61 | 0 | 92.61 | 3.33333 | 111.1321 |
| EC |  |  |  | 0 | 30 | 0 | 30 | 100 | 0 | 100 | 3.33333 | 120 |
|  |  |  |  |  |  |  |  |  |  |  |  |  |
| code | IC50 | conc | log | %inh | T2 | T1 | ∆T | RFU2 | RFU1 | ∆RFU | slope | K.Activity |
| **2p** |  | 100 | 2 | 92 | 30 | 0 | 30 | 8.04 | 0 | 8.04 | 3.33333 | 9.64801 |
|  |  | 10 | 1 | 80 | 30 | 0 | 30 | 19.69 | 0 | 19.69 | 3.33333 | 23.62802 |
|  |  | 1 | 0 | 58 | 30 | 0 | 30 | 41.82 | 0 | 41.82 | 3.33333 | 50.18405 |
|  |  | 0.1 | -1 | 47 | 30 | 0 | 30 | 53.05 | 0 | 53.05 | 3.33333 | 63.66006 |
|  |  | 0.01 | -2 | 36 | 30 | 0 | 30 | 64.31 | 0 | 64.31 | 3.33333 | 77.17208 |
| EC |  |  |  | 0 | 30 | 0 | 30 | 100 | 0 | 100 | 3.33333 | 120 |
|  |  |  |  |  |  |  |  |  |  |  |  |  |
| code | IC50 | conc | log | %inh | T2 | T1 | ∆T | RFU2 | RFU1 | ∆RFU | slope | K.Activity |
| **8p** |  | 100 | 2 | 90 | 30 | 0 | 30 | 9.58 | 0 | 9.58 | 3.33333 | 11.49601 |
|  |  | 10 | 1 | 75 | 30 | 0 | 30 | 24.73 | 0 | 24.73 | 3.33333 | 29.67603 |
|  |  | 1 | 0 | 44 | 30 | 0 | 30 | 56.39 | 0 | 56.39 | 3.33333 | 67.66807 |
|  |  | 0.1 | -1 | 30 | 30 | 0 | 30 | 69.52 | 0 | 69.52 | 3.33333 | 83.42408 |
|  |  | 0.01 | -2 | 16 | 30 | 0 | 30 | 84.42 | 0 | 84.42 | 3.33333 | 101.3041 |
| EC |  |  |  | 0 | 30 | 0 | 30 | 100 | 0 | 100 | 3.33333 | 120 |
|  |  |  |  |  |  |  |  |  |  |  |  |  |
| code | IC50 | conc | log | %inh | T2 | T1 | ∆T | RFU2 | RFU1 | ∆RFU | slope | K.Activity |
| **SAHA** |  | 10 | 1 | 91 | 30 | 0 | 30 | 9.46 | 0 | 9.46 | 3.333 | 11.353 |
|  |  | 1 | 0 | 75 | 30 | 0 | 30 | 25.41 | 0 | 25.41 | 3.333 | 30.495 |
|  |  | 0.1 | -1 | 62 | 30 | 0 | 30 | 37.81 | 0 | 37.81 | 3.333 | 45.377 |
|  |  | 0.01 | -2 | 45 | 30 | 0 | 30 | 54.86 | 0 | 54.86 | 3.333 | 65.839 |
| EC |  |  |  | 0 | 30 | 0 | 30 | 100 | 0 | 100 | 3.33333 | 120 |
|  |  |  |  |  |  |  |  |  |  |  |  |  |

HDAC2

HDAC8

HDAC8

HDAC4

HDAC4

HDAC6

**Detailed results of cell cycle analysis of compound 5d on Du-145 cells**

**Detailed results of cell cycle analysis of compound 5d on PC-3 cells**

**Detailed results of cell cycle analysis of compound 5d on MG- 63 cells**

**Detailed results of cell apoptosis of compound 5d on MG- 63 cells**

**Detailed results of cell apoptosis of compound 5d on PC-3 cells**

**Detailed results of cell apoptosis of compound 5d on Du-145 cells**
